# Cytotoxic Labdane Diterpenes, Norlabdane Diterpenes and Bis-Labdanic Diterpenes from the Zingiberaceae: A Systematic Review

**DOI:** 10.3390/ph15121517

**Published:** 2022-12-05

**Authors:** Kelvin Jianmin Voon, Yasodha Sivasothy, Usha Sundralingam, Aicha Lalmahomed, Asly Poh-Tze Goh

**Affiliations:** School of Pharmacy, Monash University Malaysia, Jalan Lagoon Selatan, Bandar Sunway 47500, Selangor, Malaysia

**Keywords:** Zingiberaceae, labdane diterpenes, norlabdane diterpenes, bis-labdanic diterpenes, cytotoxicity

## Abstract

Over the years, labdane diterpenes, norlabdane diterpenes, and bis-labdanic diterpenes with cytotoxic activities have been identified across various families in the plant kingdom including the Zingiberaceae. The present review discusses the distribution of these labdane-type diterpenes within the Zingiberaceae; their extraction, isolation, and characterization from the respective Zingiberaceae species; the structural similarities and differences within each group and between the different groups of the labdane-type diterpenes; and their cytotoxic activities against breast, cervical, liver, colorectal, pancreatic, lung and prostate cancer cell lines. The review will also provide insight into how the cytotoxic activities of the labdane-type diterpenes are influenced by their structural features.

## 1. Introduction

Cancer is a group of diseases characterized by uncontrollable cell growth. The World Health Organization (WHO) estimated in 2019 that cancer was the number one or number two cause of death among populations under the age of 70 in countries of all income levels [1]. In 2020, the total number of estimated cases was 19.3 million as compared to 14.1 and 10.9 million estimated cases in 2012 and 2002, respectively, and according to GLOBOCAN, this figure is projected to continue rising and reach an estimate of 23.4 million cases in the year 2040. The number of estimated deaths due to cancer in the years above also revealed an upward trend; 6.7 million, 8.2 million, and 10.0 million deaths were reported, respectively [1,2,3,4,5,6].

The causes of cancer can be grouped into two categories, namely internal factors and external factors. Internal factors include genetic mutations, weakened immune system and abnormal hormone levels, while external factors include smoking, alcohol abuse, diet, sedentary lifestyle, lack of physical exercise, lack of social contact, chronic stress, pollutants, and radiation [7,8,9,10,11,12,13].

Different types of treatment are available depending on the type of cancer and how advanced it is. Surgery, chemotherapy, radiation therapy, and proton therapy are conventional treatment strategies. Photodynamic therapy, photothermal therapy, gene therapy, nanoparticle-drug therapy, targeted therapy, and immunotherapy on the other hand are methods which have emerged as recently developed treatment strategies. A disadvantage of the currently available treatments, such as surgery, is the pain it causes and the potential damage to nearby tissues. Certain chemotherapy drugs can cause unintentional damage to other otherwise healthy organs. Parts of the body most commonly affected by chemotherapy drugs include bone marrow, hair follicles, and the reproductive system. There are also risks of rejection or infection from stem-cell or bone marrow transplant, and the whole process might take a long time. In addition, the costs of these treatments can be prohibitive, especially for patients from developing countries [14,15].

The currently available treatment strategies for cancer are not perfect, and there continues to be a need for more research to be carried out in order to increase the efficacy and reduce the side effects of cancer treatment. Natural anticancer agents, especially those isolated from the plant kingdom, can be a great alternative to otherwise more expensive drugs used for treating cancer. Approximately, 60% of the drugs in use today in cancer therapy were first obtained from various natural sources, and of those, many were sourced from varying species in the Kingdom Plantae [16].

The plant kingdom is a rich source of phytochemicals which cover a wide and diverse range of products, among which include alkaloids, terpenoids, flavonoids, and other types of polyphenolic compounds. These plant-derived products possess a broad spectrum of pharmacological activities which enable them to play important roles in the prevention or treatment of many diseases, including cancer [10,16]. Vincristine is an example of an anticancer agent sourced from plants, namely from the leaves of *Catharanthus roseus* (L.) G. Don, that has been used effectively in treating various cancers such as leukemia, lymphoma, breast cancer, and more. Vinblastine, another anticancer agent extracted alongside vincristine from the same source, is also effective in battling similar types of cancers as vincristine [17]. These vinca alkaloids disrupt microtubules, causing the arrest of the cells at metaphase, which leads to apoptotic cell death [18]. Another example of an anticancer drug derived from plants is paclitaxel, which is extracted from the bark of *Taxus brevifolia* Nutt., and it is used extensively in the treatment of a variety of cancers, including ovarian cancer, breast cancer, and lung cancer [19]. This taxane diterpenoid promotes the polymerization of tubulin heterodimers to microtubules, in turn suppressing the dynamic changes in microtubules, thus resulting in mitotic arrest [18].

The Zingiberaceae, commonly known as the ginger family, is one of the largest families of the plant kingdom [20]. It is a family of perennial aromatic flowering plants with creeping horizontal or tuberous rhizomes. It comprises 53 genera and about 1300 species distributed throughout tropical Africa, Asia, and the Americas. Many of these species have been used for centuries as remedies in various folk or traditional medicinal systems around the world to treat a wide range of ailments. Plants of the Zingiberaceae have also been extensively used as spices or flavoring agents, due to their characteristic organoleptic properties (color, pungent and spicy taste, and aromatic odor) [21,22,23].

Phytochemical investigation of the Zingiberaceae has led to the isolation and characterization of various classes of secondary metabolites such as essential oils, flavonoids (e.g., flavanones, flavonols, chalcones, and flavan-3-ols), terpenoids (e.g., monoterpenoids, sesquiterpenoids, diterpenoids, and triterpenoids), diarylalkanoids or arylalkanoids (e.g., diarylheptanoids and diarylpentanoids), phenylpropanoids (e.g., phenylbutenoids) and phenolics (e.g., lignans, coumarins, and phenolic acids). These secondary metabolites exhibit numerous pharmacological activities, namely anticancer, antioxidant, antimicrobial, antiviral, anti-inflammatory, anti-allergic analgesic, hypoglycemic, neuroprotective, cardiovascular-protective, and digestive-system-protective activities [24,25,26,27,28,29].

The current paper reviews the published scientific literature related to the cytotoxic activities of the secondary metabolites isolated from the Zingiberaceae, with the emphasis being on the labdane-type diterpenes, a sub-class of the diterpenoids. This review aims to provide insight into the cytotoxic potential of the labdane-type diterpenes isolated and characterized from the Zingiberaceae that may have the potential to be developed into safe and affordable chemotherapy drugs. This review will act as a database and a foundation for further research on cytotoxic labdane-type diterpenes, which were not only isolated from the Zingiberaceae but also other families in the plant kingdom.

## 2. Methods

This systematic review was carried out according to the guideline requirements of the Preferred Reporting Items for Systematic Reviews and Meta-Analyses (PRISMA). The authors K.J.V. and A.L. independently performed the data extraction and data synthesis for this systematic review using the PICO framework (Population, Intervention, Comparison, and Outcome). Studies applicable to the aim of this review were searched using two electronic databases: Web of Science—Core Collection and SciFinder. “Labdane diterpenes” and “cytotoxic” were the two keywords which were used to search the electronic databases for relevant articles. The complete list of keywords which were used in the search is presented in Table 1. Each term was used in both its singular and plural forms. The search was limited to looking for the keywords in the title and the abstract of the articles. Only articles published between the year 2000 and the time of the execution of the search (28 March 2022) were taken into consideration for the preparation of this review.

Articles were selected based on the labdane-type diterpenes isolated and characterized from the Zingiberaceae that exhibited cytotoxic activity. Only scientific research articles, including short communications, notes, and letters, written in English and published between the year 2000 and 28 March 2022 that documented the IC_50_ and GI_50_ values of the in vitro cytotoxic activities were reported in this review. Studies which compared the cytotoxic activities of the naturally occurring labdane-type diterpenes with a comparator or a standard drug, or a positive control were included. Scientific research papers related to human trials, clinical trials, randomized control trials, animal studies, literature reviews, systematic reviews, meta-analyses, conference proceedings, and patents were excluded from this review. In addition, studies that did not fully report on the results or methodologies and studies in which the full text was not accessible were not included in this review.

Based on the inclusion and exclusion criteria presented in Table 2, title, abstract and full-text screening were performed independently by two reviewers (K.J.V. and A.L.) via COVIDence (https://COVIDence.org/) accessed on 14 April 2022. Cases of disagreement were resolved by another independent reviewer (Y.S.) to prevent biases.

## 3. Results

**Figure 1:**
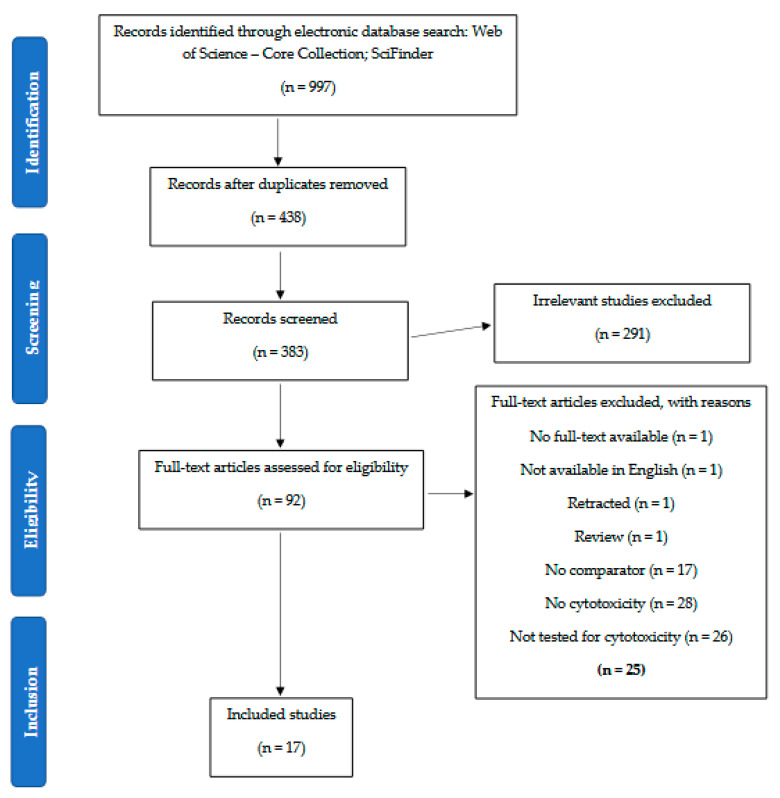
PRISMA flow chart for article selection.

A total of 997 articles were found from the search conducted using the two electronic databases. The articles were transferred into EndNote, following which the duplicates were removed and 406 articles remained. The references to these articles were then uploaded to COVIDence, where an additional 23 duplicates were removed, leaving 383 articles. The articles were first screened using their title and abstract in COVIDence, during which 291 articles were excluded, and 92 articles were left. The 92 articles were then further screened through full-text screening, where 75 articles were excluded. The reasons for the exclusion of certain articles are depicted in Figure 1. Finally, 17 articles specifically focusing on the identification of cytotoxic labdane-type diterpenes from the Zingiberaceae were considered relevant for inclusion in this review and thus were selected for an in-depth analysis. The schematic PRISMA flow diagram of the search and the process of inclusion of the articles is depicted in Figure 1. The results of the search are summarized in Table 3, Table 4, Table 5, Table 6, Table 7, Table 8, Table 9 and Table 10, which highlight the main findings obtained from our analyses of the 17 articles.

## 4. Discussion

### 4.1. Phytochemical Investigation

The chemical structures of the cytotoxic labdane-type diterpenes and their names in relation to the corresponding Zingiberaceae species are illustrated in Figure S1 and presented in Table 3, respectively. Table S1 summarizes the techniques which were used to extract, isolate and characterize the cytotoxic labdane-type diterpenes from the respective Zingiberaceae species.

#### 4.1.1. Distribution of the Cytotoxic Labdane-Type Diterpenes within the Zingiberaceae

A total of forty-four cytotoxic labdane-type diterpenes have been identified in the Zingiberaceae till the present. These compounds were obtained from thirteen different species from the following five genera: *Alpinia* (*A. calcarata* Rosc. and *A. intermedia* Gagnep)*, Amomum* (*A. maximum* Roxb)*, Curcuma* (*C. mangga* Val. van Zip; *C. mutabilis* Škorničk., and M. Sabu & Prasanthk.)*, Hedychium* (*H. coronarium* J. Koenig; *H. ellipticum* Buch.-Ham. ex Sm.; *H. forrestii* Tong.; *H. gardnerianum* Sheppard ex Ker Gawl; *H. longipetalum* X. Hu & N. Liu; *H. spicatum* Buch.-Ham. ex Sm.; and *H. yunnanense* Gagnep.)*,* and *Roscoea* (*R. purpurea* Sm.) (Table 3). Among these, thirty-four compounds were isolated and characterized from the genus *Hedychium*: three compounds each from *Alpinia* and *Roscoea* and two compounds each from *Amomum* and *Curcuma* (Table 3).

#### 4.1.2. Extraction, Isolation, and Characterisation of the Cytotoxic Labdane-Type Diterpenes

The labdane-type diterpenes were extracted from either the rhizomes, roots, leaves, or the aerial parts of the different Zingiberaceae species using the solvent extraction method. The extraction of the plant materials was carried out either at room temperature or under reflux using organic solvents or aqueous alcohol (Table S1).

The resulting crude solvent extracts were either used directly or further partitioned with organic solvents and subjected to various chromatographic techniques, mainly silica gel column chromatography, preparative TLC, and preparative HPLC, to isolate and purify the labdane-type diterpenes (Table S1).

The structures of these labdane-type diterpenes were subsequently elucidated by a combination of various spectroscopic techniques such as infrared spectroscopy (IR), ultraviolet-visible spectroscopy (UV-Vis), mass spectrometry (MS) and nuclear magnetic resonance spectroscopy (NMR) (Table S1).

#### 4.1.3. Structures of the Cytotoxic Labdane-Type Diterpenes

Among the forty-four cytotoxic labdane-type diterpenes identified through this search, forty are characterized as labdane diterpenes (**3–27**, **29–37**, **39–44**), two as norlabdane diterpenes (**28**, **38**) and two as bis-labdanic diterpenes (**1**, **2**) (Table 3 and Figure S1). The following sections will discuss the structural similarities and differences within each group of the labdane-type diterpenes and between the different groups of the labdane-type diterpenes.

##### Labdane Diterpenes

All of the labdane diterpenes in the present study consist of twenty to twenty-four carbon atoms. Their basic framework is constructed from two substructures: a decalin moiety (C-1-C-10) which consists of two fused cyclohexane rings and a branched six-membered carbon side chain (C-11-C-16; with C-13 attached to C-16) which is connected to position C-9 of the decalin moiety [47]. The three tertiary methyl groups (C-18, C-19, and C-20) are bonded to positions C-4 and C-10 of the decalin moiety (Figure S2).

The C-17 group which is bonded to position C-8 of the labdane framework varied between the structures. There are four different types. A majority of the labdane diterpenes (**3–17**, **20**, **21**, **24–27**, **29–36**, **43**, **44**) have an exomethylene group at their position C-8. As for the remaining labdane diterpenes, either a methyl (**22**, **23**, **40–42**), aldehyde (**18**, **37**) or hydroxymethyl (**19, 39**) group occupied their C-8 position instead (Figure S1).

Variations were also observed in the structure of the decalin moiety. In compounds **18**, **22**, **23**, **39**, **41**, and **42**, there is a double bond between positions C-7 and C-8 and a carbonyl group at position C-6 of their decalin moieties, while in compounds **19**, **37**, and **40**, there is a hydroxyl group at position C-7 in addition to the double bond between C-7 and C-8 and the carbonyl group at C-6. As for compounds **8**, **14**, **24**, **32–36**, **43,** and **44**, either position C-6 or C-7 bears a hydroxyl group without the presence of a double bond between positions C-7 and C-8 of their decalin moieties. Compound **4** on the other hand is the only labdane diterpene which bears a carbonyl group at position C-3 of its decalin moiety. As for compounds **3**, **5–7**, **9–13**, **15–17**, **20**, **21**, **25–27**, and **29–31**, there are either no double bonds within their decalin moieties, and neither are any of the carbon atoms in their decalin moieties occupied by oxygenated functional groups (Figure S1).

It is also evident from this review that there are two distinct types of the C-11-C-16 side chain. In compounds **3**, **4**, **6**, **7**, **10–27**, **29**, **30**, **32–37**, and **39–44**, their side chains bear either a furan (**3**, **14**, **22**, **27**, **32**, **37**, **39**, **41**, and **43**), a γ-lactone (**4**, **10**, **11**, **13**, **17**, **20**, **24**, and **36**) an α,β-unsaturated γ-lactone (**6**, **12**, **16**, **18**, **19**, **21**, **23**, **29**, **30**, **33–35**, **40**, **42**, and **44**), an epoxide (**7**), an unsaturated oxane (**15**), or an unsaturated 1,2 dioxepane (**25**) ring. As for compounds **5**, **8**, **9**, **26**, and **31**, none of their side chains have a ring; instead, they have a combination of one of the following functional groups: carboxylic acid, aldehyde, hydroxymethyl, hydroxyl and ether (Figure S1).

The relative stereochemistry of the group of atoms at C-4, C-5, C-9, and C-10 for all of the labdane diterpenes are essentially the same on the basis of their biogenetic pathway. The C-18 methyl group has an equatorial orientation about position C-4 while the C-19 and C-20 methyl groups, along with the H-5 and H-9 atoms, each is axially oriented about positions C-4, C-10, C-5, and C-9, respectively (Figure S1).

##### Norlabdane Diterpenes

In general, the basic framework and the relative stereochemistry for these compounds are similar to those of the labdane diterpenes, with the only difference being in the nature of the side chain at position C-9 of the decalin moiety. For compounds **28** and **38**, instead of having a branched, six-membered carbon side chain, these compounds have either a three-membered (**38**) or a four-membered (**28**) aliphatic chain, in turn resulting in these compounds having fewer than 20 carbon atoms in their respective structures (Figure S1).

A closer look at the structures of the individual norlabdane diterpenes revealed major differences. Their decalin moieties are notably different. For compound **38**, there is a double bond between positions C-7 and C-8 and a carbonyl group at position C-6 of its decalin moiety. In contrast, the aforementioned functional groups are absent in the structure of compound **28**. The side chain at position C-9 of compound **28** is a butenone chain (-CH=CH-C(O)-CH_3_) while that of compound **38** is a propenoic acid chain (-CH=CH-COOH). In compound **28**, its C-17 group is an exomethylene group, while in compound **38**, it is a methyl group instead (Figure S1).

##### Bis-Labdanic Diterpenes

The two bis-labdanic diterpenes (**1** and **2**) which were identified in *A. calcarata* through this study are a pair of stereoisomers. Their structures are identical to one another with the exception of the orientation of the H-9 and H-9′ atoms at the corresponding C-9 and C-9′ positions of their respective decalin moieties. It is interesting to note that compounds **1** and **2** are non-symmetrical bis-labdanic diterpenes as each compound is constructed from two different monomers. Both monomers in each compound are linked together via an ether linkage (Figure S1).

### 4.2. Cytotoxic Activities

All forty-four labdane-type diterpenes included in this review were assayed against various cell lines from different types of cancers (Table 4, Table 5, Table 6, Table 7, Table 8, Table 9 and Table 10). The assays were conducted using either one of the following methods: MTT, REMA, or SRB. Eight studies performed cytotoxic assays against breast cancer cell lines, eight against cervical cancer cell lines, seven against lung cancer cell lines, five against liver cancer cell lines, five against colorectal cancer cell lines, and one each against pancreatic and prostate cancer cell lines. The cytotoxic activities of the included labdane-type diterpenes from the Zingiberaceae were analyzed using two parameters, namely the IC_50_ and GI_50_ values. IC_50_ is the half-maximal inhibitory concentration, which represents the concentration of a compound where the biological process is reduced by half, whereas GI_50_ is the half-maximal inhibition of cell proliferation, which represents the concentration of a compound where the total cell growth is reduced by 50%. The labdane-type diterpenes with IC_50_ values or GI_50_ values < 1 µM were considered potent, between 1 and 10 µM were considered strong, between 10 and 30 µM were considered moderate, and between 30 and 100 µM were considered weak (Table 4, Table 5, Table 6, Table 7, Table 8, Table 9 and Table 10). Labdane-type diterpenes whose IC_50_ values or GI_50_ values were >100 µM were considered inactive and, therefore, were not included in Table 4, Table 5, Table 6, Table 7, Table 8, Table 9 and Table 10. However, if the IC_50_ value or GI_50_ value of a labdane-type diterpene was greater than 100 µM but comparable to the IC_50_ value or GI_50_ value of the positive control, its potency was included in Table 4, Table 5, Table 6, Table 7, Table 8, Table 9 and Table 10 and is further discussed. Structure–activity relationship (SAR) studies were only discussed wherever possible for the active labdane-type diterpenes, i.e., for the labdane-type diterpenes which exhibited cytotoxic activities with IC_50_ values < 100 µM against a particular cell line.

#### 4.2.1. Breast Cancer Cell Lines

The studies included in this review carried out cytotoxic assays against the following breast cancer cell lines: MCF-7, T-47D, and MDA-MB-231 (Table 4, Table 5 and Table 6).

##### MCF-7 Cell Line

In 2014, Luo and his coworkers isolated and characterized three new labdane diterpenes (amomax A-B and **4**) and two known labdane diterpenes (ottensinin and **5**) from the dichloromethane soluble fraction of the aqueous ethanolic extract of the roots of *A. maximum.* When this group of researchers examined the cytotoxicity of these labdane diterpenes against the MCF-7 cell line using the MTT assay, they found compound **4** to be two times more effective (IC_50_ = 29.80 µM) compared to 5-fluorouracil (IC_50_ = 59.96 µM), the synthetic positive control which was used in their study [32].

According to an investigation carried out by Abas et al. back in 2005, compound **6**, which they had identified in the ethyl-acetate-soluble fraction of the acetone extract of the rhizomes of *C. manga*, was found to be a potent inhibitor of the cytotoxic activity of the MCF-7 cell line with an IC_50_ value of 0.59 µM [33]. Demethoxycurcumin (IC_50_ = 18.51 µM), bisdemethoxycurcumin (IC_50_ = 33.78 µM), and curcumin (IC_50_ = 10.44 µM) were also isolated alongside compound **6** and assessed for their potential in inhibiting the cytotoxic activity of the same cell line. These compounds were not as cytotoxic as compound **6 [33]**. A new labdane diterpene glucoside, curcumanggoside, along with compounds **26** and **31**, was also identified in the rhizomes of *C. mangga*. However, these labdane-type diterpenes were not evaluated for their cytotoxicity against the MCF-7 cell line. A separate study conducted ten years later reported a weaker cytotoxicity (IC_50_ = 39.71 µM) of compound **6** which was obtained from a different source: the rhizomes of *H. ellipticum* [39]. The difference in the IC_50_ values of compound **6** between both studies could be attributed to the differences in the assay procedures performed by the respective group of researchers. Abas et al. investigated the cytotoxicity of compound **6** using the MTT assay, while Songsri and Nuntawong employed the REMA technique instead [33,39]. Furthermore, MCF-7 is a cell line that is composed of different phenotypes with varying chromosomal numbers that can affect the cells’ gene and receptor expressions. Therefore, the variation within the MCF-7 cell lines could also have contributed to the difference in the IC_50_ values in both of the studies [48].

The hexane and dichloromethane extracts of the rhizomes of *H. ellipticum,* in a phytochemical investigation carried out by Songsri and Nuntawong, yielded ten compounds in total: eight labdane diterpenes (**6**, **16**, **20**, **27**, **29**, **30**, **31** and zerumin A) and two norlabdane diterpenes (**28** and (*E*)-14,15,16-Trinorlabda-8(17),11-dien-13-oic acid). According to the results obtained from the REMA assay, only five of these compounds (**6**, **16**, **20**, **29**, and **31**) were cytotoxic against the MCF-7 cell line. Except for compound **16** (IC_50_ = 8.75 µM) which had an IC_50_ value comparable with that of doxorubicin (IC_50_ = 1.22 µM), the cytotoxicity of the rest of the compounds was considered to be either moderate (**29**) or weak (**6**, **20**, **31**) [39]. The rather weak cytotoxicity of compounds **20** and **31** upon comparison to compounds **6**, **16**, and **29** implied that the presence of an α,β-unsaturated γ-lactone ring in the side chain played an essential role in mediating the cytotoxic activity against the tested cell line. When the cytotoxicity of compounds **16** and **29** was compared to compound **6**, the greater activity of compounds **16** and **29** led to the assumption that the potency of both these compounds could have resulted from the presence of a *trans* double bond which is conjugated to the α,β-unsaturated γ-lactone ring in their respective side chains, in contrast to compound **6** in which the *trans* double bond is absent. The variation in the α,β-unsaturated γ-lactone rings of compounds **16** and **29** could further explain the significant difference in the cytotoxicity between both of these labdane diterpenes. The carbonyl group being at position C-15 of the α,β-unsaturated γ-lactone ring in compound **16**, instead of position C-16 as in compound **29**, in addition to the presence of a hydroxyl group at position C-16 of the α,β-unsaturated γ-lactone ring in compound **16** and the absence of it in compound **29**, could have increased the cytotoxicity of compound **16** to three folds of that of compound **29**.

In an investigation by Reddy and his coworkers, the chloroform extract which they had prepared from the rhizomes of *H. spicatum* exhibited a rather mild cytotoxic activity (IC_50_ = 58.08 µg/mL) against the MCF-7 cell line [44]. Phytochemical investigation of the chloroform extract followed by the in vitro cytotoxic evaluation of the secondary metabolites isolated and characterized from the extract provided justification that the two new labdane diterpenes (**40** and **41**) along with the known labdane diterpene (**32**), although reported to be weakly cytotoxic against the MCF-7 cell line, could have been responsible for the extract’s cytotoxicity. Reddy et al. concluded that upon comparing the activity of compound **40** with that of compounds **32** and **41**, the presence of an α,β-unsaturated γ-lactone ring in the side chain along with the presence of a double bond between positions C-7 and C-8 and a carbonyl group at position C-6 of the decalin moiety of compound **40** enhanced its cytotoxicity against the tested cell line. They further deduced that even with the presence of a double bond between positions C-7 and C-8 and a carbonyl group at position C-6 of the decalin moiety, the cytotoxicity in compound **41** was decreased due to the absence of the α,β-unsaturated γ-lactone ring. The remaining labdane diterpenes, hedychilactone B and hedychilactone C—which were also identified in the chloroform extract—were found to be inactive [44].

A year ago, Singamaneni et al. prepared a chloroform fraction from the methanol extract of the rhizomes of *R. purpurea* and assessed its cytotoxicity against the MCF-7 cell line. The results obtained from their MTT assay revealed that the chloroform fraction was moderately cytotoxic (IC_50_ = 46.64 µg/mL) and slightly stronger in its activity compared to the methanol extract (IC_50_ = 48.96 µg/mL) itself. Purification of the chloroform fraction led to the isolation and characterization of three weakly cytotoxic labdane diterpenes, **14**, **43**, and **44**, with the latter two compounds being identified for the first time in the plant kingdom [46].

In 2010, Suresh et al. employed the SRB assay to compare the growth-inhibitory properties of compounds **10**, **18–24** which they had isolated and characterized from the hexane extract of the rhizomes of *H. coronarium* against the MCF-7 cell line. The results revealed that the order of their growth-inhibitory activity was **19** > **22** > **23** > **20** > **10** > **21** > **24** > **18 [36]**.

##### MDA-MB-231 and T-47D Cell Lines

In 2009, Chimnoi et al. reinvestigated the chemical constituents of the dichloromethane extract of the rhizomes of *H. coronarium*. Their investigation led to the identified two positional isomers (**17** and **20**) differing in the position of the hydroxyl and carbonyl groups in the γ-lactone-rings of their respective labdane framework. A new labdane diterpene, 7β-hydroxy-(*E*)-labda-8(17),12-diene-15,16-dial, along with known compounds (coronarin F, **25**, **28** and **31)**, were also isolated and characterized alongside the positional isomers. Chimnoi and his coworkers subsequently determined the cytotoxicities of both the positional isomers against the MDA-MB-231 cell line through the MTT assay. Compounds **17** and **20** were both considered to be equally strong inhibitors (IC_50_ < 8 µM) of the cytotoxic activity of the MDA-MB-231 cell line [37].

Compared to the strong cytotoxic activity against the MDA-MB-231 cell line, compound **20** reported less than half of that activity against the T-47D cell line, while compound **17** reported almost equal cytotoxicity against the T-47D cell line, albeit to a slightly lesser degree [37]. The difference in the reported IC_50_ values of compounds **17** and **20** against both the MDA-MB-231 and T-47D cell lines could be attributed to the fact that the two cell lines belong to different subtypes of breast cancer: basal-type and luminal-type, respectively. These subtypes of breast cancer are associated with different gene expressions and, generally, patients afflicted with basal-type breast cancers have a worse prognosis when compared to patients suffering from luminal-type breast cancers [49].

Recently, in 2021, Soumya et al. used the MTT assay to evaluate the various solvent extracts (petroleum ether, chloroform, acetone, and methanol) of the rhizomes of *C. mutablis* for their cytotoxicity against the MDA-MB-231 cell line [34]. All of the extracts exhibited a dose-dependent reduction in cell survival percentage, with the petroleum ether extract demonstrating the maximum cytotoxicity of 5.6 µg/mL. The bioassay-guided isolation of the petroleum extract led to the identification of a novel labdane diterpene (**7**) which was found to be responsible for the activity of the petroleum ether extract. Compound **7** (IC_50_ = 7.71 µM) was strongly cytotoxic and four-fold more potent compared to that of the positive control, curcumin (IC_50_ = 31.22 µM), a diarylheptanoid [34,37].

#### 4.2.2. Cervical Cancer Cell Lines

The cervical cancer cell lines KB, SMMC-7721, SGC-7901, and HeLa were used to perform the cytotoxic assays in the studies included in this review. KB, SMMC-772,1 and SGC-7901 were originally attributed as oral, liver, and gastric cancer cell lines in the studies obtained through our search. However, these cell lines have recently been proven to have been misidentified and are actually derivatives of the HeLa cell line [50,51,52,53]. Hence, in the current review, the results related to these three cell lines are included alongside the HeLa cell line as cervical cancer cell lines (Table 7, Table 8, Table 9 and Table 10). Since the KB, SMMC-7721, and SGC-7901 cell lines have been identified as cell lines that have been contaminated by HeLa cells, the cell lines have almost identical STR profiles to the HeLa cell line. However, there also exist some differences in the STR profiling of the contaminated cell lines compared to that of the Hela cells, and mutations in the microsatellites have been observed to affect certain biological functions [54,55]. These differences might have led to the discrepancies in the reported IC_50_ values between the cell lines.

##### KB Cell Lines

In 2002, Kong et al., isolated and characterized two novel stereoisomeric bis-labdanic diterpenes (**1** and **2**) from the ethyl acetate fraction of the methanol extract of the rhizomes of *A. calcarata*. Based on the results obtained from their MTT assay, both compounds **1** (IC_50_ = 0.34 µM) and **2** (IC_50_ = 0.24 µM) can be regarded as potent inhibitors of the cytotoxic activity of the KB cell lines due to their IC_50_ values being significantly comparable to the positive control, adriamycin (IC_50_ = 0.15 µM) [30].

In addition to the MCF-7 cell line, Songsri and Nuntawong also assessed the cytotoxic potentials of the ten labdane-type diterpenes which they had identified in the hexane and dichloromethane extracts of the rhizomes of *H. ellipticum* against the KB cell line. Only six of them were active. For compound **16** (IC_50_ = 2.75 µM), although its cytotoxic activity was strong and almost comparable to ellipticine (IC_50_ = 1.23 µM), it was found to be slightly weaker than that of doxorubicin (IC_50_ = 0.22 µM) [39]. As for the cytotoxic activity of compounds **6**, **27**, **29**, **30**, and **31**, these compounds can be regarded as being either moderate (**29**) or weak (**6**, **27**, **30**, **31**) [39]. The same structural features of compounds **16** and **29**, which could have given rise to their potency against the MCF-7 cell line as discussed in Section MCF-7 Cell Line, may also be responsible for their potency against the KB cell line. Although compound **30** is almost identical in structure to compound **29**, the presence of a methoxyl group at position C-15 of its α,β-unsaturated γ-lactone ring could have considerably suppressed its activity by five-fold in comparison to compound **29**.

Chimnoi et al. also determined the cytotoxicities of the two positional isomers (**17** and **20**) isolated and characterized from the rhizomes of *H. coronarium* against the KB cell line through the MTT assay. Their findings revealed that both compounds exhibited strong cytotoxic activity (IC_50_ = 8.81 and 9.43 µM, respectively) against the KB cell line with similar inhibiting potentials [37].

##### SMMC-7721 and SGC-7901 Cell Lines

With regard to the cytotoxic activity of the SMMC-7721 cell line, compound **4** (IC_50_ = 24.38 µM) from the roots of *A. maximum* was once again identified by Lou and his coworkers as a more effective inhibitor (three folds) compared to the synthetic positive control, 5-fluorouracil (IC_50_ = 77.64 µM). The cytotoxicity of the remaining labdane diterpenes identified in this plant was considered to be inactive against this cell line [32].

Li and his coworkers in 2016 isolated and characterized two new labdane diterpenes (hedychenoid A and **42**) along with four known labdane diterpenes (forrestin A, calcaratarin C, **22** and **29**) from the ethyl acetate fraction of the ethanol extract of the rhizomes of *H. yunnanense*. According to the results from their SRB assay, compounds **22** and **29** exhibited moderate cytotoxic activities against the SGC-7901 cell line with similar inhibiting strengths. Compound **42**, on the other hand, was a weaker inhibitor. The remaining labdane diterpenes were considered inactive [45]. Although compounds **22** and **42** possessed identical decalin moieties, with each compound having a double bond between positions C-7 and C-8, a carbonyl group at position C-6 and a methyl group at position C-8, the variation in their respective side chains could have attributed to the differences in their potency against the SGC-7901 cell line. The presence of a furan ring in compound **22** could have increased its activity by almost two folds compared to compound **42** which instead has an α,β-unsaturated γ-lactone ring bearing a methoxyl group at position C-15.

In a separate study, Zhao et al. isolated and characterized three new labdane diterpenes (hedylongnoids A-B and **36**) together with three known ones (**32**, **34**, and **35**) from the rhizomes of *H. longipetalum* [42]. The SRB assay was used to determine the cytotoxic potentials of these isolates against the SGC-7901 cell line, and among them, compounds **32**, **35**, and **36** were moderately cytotoxic, with the former two exhibiting similar cytotoxic strength [42]. As for compound 34, it was only weakly cytotoxic against the SGC-7901 cell line. The remaining compounds were considered to be inactive [42]. Despite compounds **34** and **35** being structurally similar to one another, the presence of the carbonyl group at position C-15 along with the hydroxyl group at position C-16 of the α,β-unsaturated γ-lactone ring in compound **35** may have increased its potency against the SGC-7901 cell line by two-fold in comparison to compound **34**, whose carbonyl group is at position C-16 and lacks a hydroxyl group in its α,β-unsaturated γ-lactone ring.

##### HeLa Cell Line

Chimnoi et al. in their investigation also evaluated the cytotoxicities of 7β-hydroxy-(*E*)-labda-8(17),12-diene-15,16-dial, **17**, **20** and **25** from the rhizomes of *H. coronarium* against the HeLa cell line. They reported that compounds **17** (IC_50_ = 9.12 µM) and **25** (IC_50_ = 8.54 µM) were strong inhibitors with almost similar ability in inhibiting the cytotoxic activity of the HeLa cell line. Compound **20**, however, was only a moderate inhibitor while the new labdane diterpene, 7β-hydroxy-(*E*)-labda-8(17),12-diene-15,16-dial, was inactive [37].

The preliminary screening of the ethyl acetate fraction of the aqueous ethanolic extract of the rhizomes of *H. coronarium* by Zhan and his coworkers using the SRB method revealed it to be cytotoxic against the HeLa cell line [35]. Subsequent purification of the crude ethyl acetate fraction in order to determine the secondary metabolites responsible for its cytotoxic activity led to the isolation and characterization of ten labdane diterpenes (**8–17**), with compounds **8** and **9** being identified as new. Only six of these labdane diterpenes were found to be cytotoxic, two of them moderately (**15** and **16**) and four of them weakly (**8**, **10**, **13**, **14**) [35]. A significant difference in the potencies was observed between compounds **10** and **13** even though both compounds were almost structurally identical to each other. The additional presence of a methoxyl group at position C-15 in the γ-lactone ring of compound **10** could have suppressed its cytotoxicity by two and a half folds compared to compound **13**. Instead, in compound **16**, when a *trans* double bond is conjugated to an α,β-unsaturated γ-lactone ring bearing a hydroxyl group at position C-16, this structural feature could have notably increased its cytotoxicity by almost four-fold compared to compound **10**.

Similarly, the SRB method was used to compare the cytotoxic potentials of the six labdane diterpenes from the rhizomes of *H. longipetalum* against the HeLa cell line [42]. In this investigation, the rhizomes yielded four cytotoxic labdane diterpenes (**32**, **34–36**). Compounds **32** and **35** were found to moderately inhibit the cytotoxic activity of the HeLa cell lines, while the remaining two compounds were less effective inhibitors [42]. The same structural features of compounds 34 and 35, which could have given rise to their potencies against the SGC-7901 cell line as discussed in Section SMMC-7721 and SGC-7901 Cell Lines, may also be responsible for their potencies against the HeLa cell line.

Suresh and his coworkers in 2010 also assessed the growth-inhibitory properties of the labdane diterpenes, which they had identified in the rhizomes of *H. coronarium* against the HeLa cell line. The results from their SRB assay showed that only compounds **18–24** weakly inhibited the growth of the HeLa cell line, while the remaining labdane diterpenes were found to be inactive (GI_50_ > 100 µM) [36].

#### 4.2.3. Liver Cancer Cell Lines

Cytotoxicity assays of compounds performed on HepG2 and HCC-S102 liver cancer cell lines are reported in this review (Table 11 and Table 12).

##### HepG2 Cell Line

In an investigation conducted by Chen et al. in 2017, the leaves of *A. intermedia* were initially extracted with 95% ethanol, following which the aqueous ethanolic extract was partitioned between hexane and water. When the aqueous ethanolic extract and the hexane fraction were evaluated for their cytotoxicity against the HepG2 cell line using the MTT assay, the hexane fraction (IC_50_ = 66.50 µg/mL) was found to be more cytotoxic compared to the aqueous ethanolic extract (IC_50_ > 100 µg/mL). The phytochemical investigation of the hexane fraction yielded two new labdane diterpenes (**3** and intermedin B) and a known labdane diterpene (**27**). Intermedin B is a C-12 epimer of **3**, and both these labdane diterpenes were structurally related to **27** (Chen et al., 2017). When Chen and his coworkers assessed the cytotoxic potential of compounds **3** and **27** towards the HepG2 cell line, they discovered that compound **3** (IC_50_ = 119.82 µM) exhibited a cytotoxic activity almost identical to that of the natural anticancer compound, andrographolide (IC_50_ = 111.94 µM), a labdane diterpene previously isolated and characterized from *Andrographis paniculata* (Burm. fil.) Nees. As for compound **27**, its cytotoxicity was almost three-fold weaker compared to compound **3** and andrographolide [31].

When Lou et al. evaluated compound **5** from the roots of *A. maximum* for its cytotoxicity towards the HepG2 cell line, the MTT assay results revealed that although compound **5** had only an IC_50_ value of 30.1 µM, it possessed stronger cytotoxic activity against the cancer cell when compared to the synthetic positive control, 5-fluorouracil, which had an IC_50_ value of more than double (IC_50_ = 76.11 µM) that of compound **5** [32].

Abas et al. also investigated the cytotoxicity of compound **6** from the rhizomes of *C. manga* against the HepG2 cell line, and it was regarded as moderately cytotoxic compared to its ability in inhibiting the activity of the MCF-7 cell line. Demethoxycurcumin (IC_50_ > 50 µM), bisdemethoxycurcumin (IC_50_ > 50 µM), and curcumin (IC_50_ = 33.53 µM) which were also identified in the rhizomes were only considered to be weakly cytotoxic against the HepG2 cell line [33].

The ethyl acetate fraction of the aqueous ethanolic extract of the rhizomes of *H. coronarium* prepared by Zhan and his coworkers was also found to be cytotoxic against the HepG2 cell line in addition to the HeLa cell line [35]. The ten labdane diterpenes (**8–17**) which were isolated and characterized from the active extract, were investigated for their cytotoxicity. Taxol, a tetracyclic diterpenoid, was used as the positive control [35]. Compounds **15**, **16**, and **17** were highly effective (IC_50_ < 10 µM) in inhibiting the cytotoxic activity of the HepG2 cells, with the former two compounds being almost equally potent inhibitors (IC_50_ = 0.9 µM and 1.1 µM, respectively) and relatively comparable to taxol (IC_50_ = 0.9 µM). The remaining compounds exhibited moderate cytotoxic activities. Compounds **13** (IC_50_ = 10.6 µM) and **14** (IC_50_ = 10.7 µM) possessed almost identical cytotoxicity, while the cytotoxic activity of compounds **8**, **9**, **10**, **11**, and **12** increased in the following order: **12** > **11** > **10** > **8** > **9** [35]. Although compounds **10**, **11**, and **13** each possessed a γ-lactone ring in their respective side chains, it is evident that the presence of either a methoxyl or ethoxyl group at position C-15 of the γ-lactone ring in compounds **10** and **11** caused a slight decrease in their respective activities compared to the activity of compound **13** in which those oxygenated functional groups are absent from position C-15 of its γ-lactone ring. Upon further comparison of the activity of compound **13** with that of compound **17**, although they are structurally similar to one another, the presence of a hydroxyl group at position C-14 of the γ-lactone ring in compound **17** slightly increased its activity compared to compound **13** in which the hydroxyl group is absent from position C-14 of its γ-lactone ring. The variation in the α,β-unsaturated γ-lactone rings of compounds **12** and **16** could explain the significant difference in the cytotoxicities between both of these labdane diterpenes. The carbonyl group being at position C-15 instead of position C-16, in addition to the presence of a hydroxyl group at position C-16 of the α,β-unsaturated γ-lactone ring in compound **16**, could have increased its cytotoxicity to almost twelve folds compared to that of compound **12** whose carbonyl group is at position C-16 instead of position C-15 and lacks a hydroxyl group in its α,β-unsaturated γ-lactone ring. It is noteworthy to mention that the unsaturated oxane ring in compound **15** could have attributed to its remarkable potency when compared to the potencies of the other compounds in which their side chains bear either a furan ring, a γ-lactone ring, an α,β-unsaturated γ-lactone ring or oxygenated functional groups.

There was a difference in the cytotoxic inhibiting potential for compound **17** according to an earlier study conducted by Chimnoi and his coworkers, who also isolated this labdane diterpene from the same plant, the rhizomes of *H. coronarium* [37]. In contrast to the results obtained by Zhan et al. in 2012, Chimnoi et al. reported that compound **17** was a moderate inhibitor with only half the cytotoxic strength [35,37]. The difference in the reported IC_50_ values between both of the studies could be attributed to the different types of assays which were used to determine the cytotoxicity of the compound. Zhan and his coworkers investigated the cytotoxicity of compound **17** using the SRB assay, while Chimnoi and his team of researchers employed the MTT method instead. Chimnoi et al. also evaluated the cytotoxic potentials of 7β-hydroxy-(*E*)-labda-8(17),12-diene-15,16-dial, **20**, and **25**. Compound **20**, unlike its positional isomer, **17**, showed a weak cytotoxicity against the HepG2 cell line, while compound **25** and 7β-hydroxy-(*E*)-labda-8(17),12-diene-15,16-dial were moderately cytotoxic and inactive, respectively [37].

##### HCC-S102 Cell Line

In addition to the HepG2 cell line, Chimnoi et al. further assessed the cytotoxicity of compounds **17** and **20** against the HCC-S102 cell line. Interestingly, although compound **17** was moderately cytotoxic towards the HepG2 cell line, yet it was only weakly cytotoxic towards the HCC-S102 cell line with almost twice the IC_50_ value with that recorded against the HepG2 cell line [37]. HepG2 is a hepatoblastoma cell line, while HCC-S102 is a hepatocellular carcinoma cell line [56,57]. The immature liver precursor cells of the hepatoblastoma cell line could have contributed to the differences in the IC_50_ values and resulted in its susceptibility to the cytotoxic activity of compound **17**. Compound **20** once again was weakly cytotoxic [37].

#### 4.2.4. Colorectal Cancer Cell Lines

Cytotoxic assays against the following colorectal cancer cell lines HCT-116, HT-29, COLO 205, and DLD-1 are included in this review (Table 13, Table 14, Table 15 and Table 16).

##### HCT-116 Cell Line

There were only two recent studies which evaluated the cytotoxicity of the labdane-type diterpenes from the Zingiberaceae against this cell line.

The first study by Soumya et al. in 2021 revealed compound **7** to be strongly cytotoxic. Compound **7** was isolated from the petroleum ether extract of the rhizomes of *C. mutablis* which demonstrated a slightly stronger cytotoxicity (IC_50_ = 5.4 µg/mL) against the HCT-116 cell line compared to the chloroform (IC_50_ = 5.5 µg/mL) and acetone (IC_50_ = 6.0 µg/mL) extracts and a far more potent cytotoxicity compared to the methanol (IC_50_ = 79.5 µg/mL) extract. The IC_50_ value of compound **7** was reported to be 3.21 µM, which was about seven times lower when compared to the positive control curcumin (IC_50_ = 21.45 µM) [34]. Compound **7** exerted its cytotoxicity towards the cancer cells by the induction of apoptosis and the suppression of genes related to cell survival and migration, while also being safe for normal cells at the same concentration [34]. Based on the investigation conducted by Soumya and her coworkers, compound **7** was identified to be 2.5-fold more effective in inhibiting the cytotoxic activity of the HCT-116 cell line compared to the MDA-MB-231 cell line [34].

In another study conducted the same year, Singamaneni et al. investigated the cytotoxic potentials of the labdane diterpenes (**14**, **43**, and **44**) from the chloroform fraction of the methanol extract of the rhizomes *R. purpurea* against the HCT-116 cell line. Compound **43** was found to moderately inhibit the cytotoxic activity of the cells while compounds **14** and **44** were only weakly cytotoxic [46]. Compounds **14** and **43** both have a hydroxyl group at position C-7 of their respective decalin moieties and a side chain bearing a furan ring. However, in compound **14**, the presence of a *trans* double bond conjugated to the furan ring and the absence of it in compound **43** could be a reason for the two folds decrease or more in the cytotoxic activity of compound **14** against the HCT-116 cell line when compared to that of compound **43**.

##### HT-29 Cell Line

In 2012, Zhan and his group of researchers examined the cytotoxic potentials of ten compounds, **8–17**, isolated and characterized from the ethyl acetate fraction of the aqueous ethanolic extract of the rhizomes of *H. coronarium* against the HT-29 cell line in addition to the HepG2 and HeLa cell lines [35]. Compounds **10** and **12** were the most cytotoxic and exhibited equal potentials in inhibiting the cytotoxic activity of the HT-29 cell line. Both compounds recorded the lowest IC_50_ value of 7.8 µM followed by compounds **17** (IC_50_ = 8.8 µM) and **11** (IC_50_ = 9.8 µM). Compounds **8**, **9**, and **13–16** on the other hand were moderately cytotoxic with relatively similar inhibitory strengths [35]. In general, the compounds with side chains that bear either a furan ring, a γ-lactone ring, an α,β-unsaturated γ-lactone ring, or an unsaturated oxane ring were slightly more effective in inhibiting the cytotoxic activity of the HT-29 cell line compared to the compounds whose side chain does not bear a ring. Although compounds **10**, **11**, and **13** each possessed a γ-lactone ring in their respective side chains, it is evident that the presence of either a methoxyl or ethoxyl group at position C-15 of the γ-lactone ring in compounds **10** and **11** resulted in an increase in their respective activities compared to the activity of compound **13** whose γ-lactone ring does not bear either one of those oxygenated functional groups. Upon further comparison of the activity of compound **13** with that of compound **17**, although they are structurally similar to one another, the presence of a hydroxyl group at position C-14 of the γ-lactone ring in compound **17** increased its activity compared to compound **13** whose γ-lactone ring does not bear a hydroxyl group at its position C-14. The variation in the α,β-unsaturated γ-lactone rings of compounds **12** and **16** could explain the difference in the cytotoxicities between both of these labdane diterpenes. The carbonyl group being at position C-15 instead of position C-16 in addition to the presence of a hydroxyl group at position C-16 of the α,β-unsaturated γ-lactone ring in compound **16** could have decreased its cytotoxicity compared to that of compound **12** whose carbonyl group is at position C-16 instead of position C-15 and lacks a hydroxyl group in its α,β-unsaturated γ-lactone ring.

##### COLO 205 Cell Line

Reddy et al. in their investigation identified the chloroform extract which they had prepared from the rhizomes of *H. spicatum* to be slightly more cytotoxic against the COLO 205 cell line (IC_50_ = 43.14 µg/mL) compared to the MCF-7 cell line (IC_50_ = 58.08 µg/mL). Once again, it would be reasonable to postulate that compounds **32**, **40**, and **41** could have contributed to the cytotoxic activity of the chloroform extract against the COLO 205 cell line like they did with regard to the MCF-7 cell line [44]. The same structural features of compounds **32**, **40**, and **41** which could have given rise to their potency against the MCF-7 cell line as discussed in Section MCF-7 Cell Line may also be responsible for their potency against the COLO 205 cell line [44].

##### DLD-1 Cell Line

In 2017, Chen et al. conducted a phytochemical investigation of the aerial part of *H. coronarium*. The hexane fraction of its methanol extract yielded hedychiumin, a new norlabdane diterpene, two known labdane diterpenes (**14** and **26**), and a known norlabdane diterpene (**28**). Among these four compounds, only compounds **14** and **26** exhibited cytotoxic activity against the DLD-1 cell line [38]. The remaining two compounds were inactive. The inactivity of the norlabdane diterpenes led to an assumption that a six-membered carbon side chain bonded to the decalin moiety is a prerequisite for the cytotoxic activity against the DLD-1 cell line. With regard to compound **14**, its cytotoxic potential towards the DLD-1 cell line, according to the study conducted by Chen et al., was found to be almost three times weaker compared to its cytotoxic potential against the HT-29 cell line from an earlier study by Zhan et al. [35,38]. Both cell lines express the p53 gene; however, the DLD-1 cells have a mutation at position 241 of the gene, while the HT-29 cells have a mutation at position 273 [58,59,60]. The mutation in the DLD-1 cells results in a phenylalanine replacing a serine, while in the HT-29 cells, an arginine is replaced by a histidine. The change in the p53 tumor antigen could have also contributed to the differences in the reported IC_50_ values.

#### 4.2.5. Pancreatic Cancer Cell Lines

A limited number of pancreatic cancer cell lines were used to study the cytotoxic activities of the labdane-type diterpenes isolated and characterized from the Zingiberaceae, with BxPC-3 being the only type of cell line to date (Table 17). Among all of the compounds identified through this review, only compounds **14**, **43**, and **44** which were isolated and characterized from the chloroform fraction of the methanol extract of the rhizomes of *R. purpurea* were recently tested for their ability in inhibiting the cytotoxic activity of the BxPC-3 cell line, and according to Singamaneni et al. who conducted the assay, only compound **14** was found to be moderately cytotoxic [46]. Based on the large difference in the cytotoxic activities between compounds **14** and **44**, it would be reasonable to conclude that the furan ring in the side chain of compound **14** could have promoted its cytotoxic activity compared to the α,β-unsaturated γ-lactone ring in the side chain of compound **44**. Although compounds **14** and **43** are structurally similar to one another, the greater activity of compound **14** in comparison to compound **43** could be due to the presence of the *trans* double bond which is conjugated to the furan ring in compound **14**, and the absence of it in compound **43**. Singamaneni and his coworkers identified compound **14** to be more effective in inhibiting the cytotoxic activity of the BxPC-3 cell line compared to those of MCF-7 and HCT-116 [46].

#### 4.2.6. Lung Cancer Cell Lines

The studies included in this review tested the cytotoxicities of the compounds against lung cancer cell lines, namely the A-549 and NCI-H187 lung cancer cell lines (Table 18 and Table 19).

##### A-549 Cell Line

The chloroform fraction (IC_50_ = 21.35 µg/mL) of the methanol extract of the rhizomes of *R. purpurea* was reported to be slightly more cytotoxic against the A-549 cell line compared to the methanol extract (IC_50_ = 25.71 µg/mL) itself [46]. According to the results obtained from the MTT assay of the labdane diterpenes isolated and characterized from the chloroform fraction, it can be inferred that compound **43** which exhibited moderate cytotoxicity could have mainly been responsible for the cytotoxic potential of the chloroform fraction [46]. A comparison between the structures of compounds **43** and **44** revealed that the presence of a furan ring in the side chain would be preferred for cytotoxic activity against the A549 cell line rather than the presence of an α,β-unsaturated γ-lactone ring conjugated with a *trans* double bond. Similarly, when the structures of compounds **14** and **43** were compared, a *trans* double bond conjugated to the furan ring in the side chain was found to weaken the activity of compound **14** by 4.5-folds compared to the activity of compound **43**.

In another investigation by Chimnoi et al., their findings enabled us to conclude that compounds **17**, **20**, and **25** from the rhizomes of *H. coronarium* demonstrated cytotoxicity against the A-549 cell line while the new labdane diterpene, 7β-hydroxy-(*E*)-labda-8(17),12-diene-15,16-dial, was inactive. Although compound **17** was only moderate in activity, it was found to be almost five times stronger in inhibiting the cytotoxic activity of the A-549 cell line compared to compounds **20** and **25** which exhibited similar inhibiting strengths one another [37].

In 2009, Reddy et al. reinvestigated the chemical constituents of the rhizomes of *H. spicatum* which they had originally investigated a year earlier [43]. In the current investigation, Reddy and his coworkers extracted the dried rhizomes with a mixture of dichloromethane: methanol (50:50 *v*/*v*) instead of using chloroform as they did in their earlier phytochemical investigation. They evaluated the cytotoxic potential of the extract against the A-549 cell line using the MTT assay and found it to be mildly cytotoxic (IC_50_ = 63.21 µg/mL). Upon further investigation of the active extract, the purification of it followed by spectroscopic characterization of the secondary metabolites obtained from it led to the identification of a new labdane diterpene (**37**) a new norlabdane diterpene (**38**), and two known labdane diterpenes (**27** and **39**). All four compounds, however, were only weakly cytotoxic with compound **37** being the most cytotoxic among the four [43]. It would be reasonable to conclude that although both compounds **27** and **37** have a *trans* double bond which is conjugated to a furan ring in their respective side chains, the presence of a double bond between positions C-7 and C-8 and a carbonyl group at position C-6 of the decalin moiety of compound **37** could have contributed to its greater cytotoxicity against the tested cell line compared to compound **27** in which there is no double bond between positions C-7 and C-8 and no carbonyl group at position C-6 of its decalin moiety. Even with the presence of a double bond between positions C-7 and C-8 and a carbonyl group at position C-6 in the respective decalin moieties of compounds **37** and **39**, the cytotoxicity of compound **39** was weaker than that of compound **37** due to the absence of a hydroxyl group at position C-7 in the decalin moiety of compound **39**. The variation in the type of labdane-type diterpenes which were identified between the previous and present studies could have been due to the following factors: collection site (different country or town), altitude, climate (humidity, rainfall, and temperature), season, soil pH, plant maturity, genotype, variety, harvesting time, postharvest handling conditions and storage, method of extraction and isolation and purification techniques [21,61,62,63,64].

The phytochemical investigation of the ethyl acetate fraction of the aqueous ethanolic extract of the rhizomes of *H. forrestii* yielded two new (hedyforrestin D and 15-ethoxy-hedyforrestin D) and three known (**32**, **33** and yunnancoronarin C) labdane diterpenes [40]. Upon evaluation of their cytotoxic potentials against the A-549 cell line using the MTT assay, only compounds **32** and **33** were reported to demonstrate the ability to inhibit the cytotoxic activity of this cell line. Among these two labdane diterpenes, the cytotoxicity of compound **33** was found to be potent (IC_50_ = 0.92 µM) and comparable to the cytotoxicity of the positive control, cisplatin (IC_50_ = 0.77 µM) [40]. Although both compounds have identical decalin moieties to one another, the differences in their respective side chains could have resulted in the significant difference in their cytotoxicities. The presence of a furan ring in the side chain of compound **32** may have decreased its potency by twelve-fold compared to compound **33** which bears an α,β-unsaturated γ-lactone ring in its side chain. As for hedyforrestin D (IC_50_ > 300.81 µM), 15-ethoxy-hedyforrestin D (IC_50_ > 277.40 µM), and yunnancoronarin C (IC_50_ > 300.81 µM), they were considered to be inactive [40].

In addition to the MCF-7 and HeLa cell lines, Suresh and his coworkers also measured the growth-inhibitory activities of the cytotoxic labdane diterpenes (**10**, **18–24**) from the hexane extract of the rhizomes of *H. coronarium* against the A-549 cell line [36]. According to the results from their SRB assay, only compounds **20–22** strongly inhibited the growth of the A-549 cell line, with compound **21** (GI_50_ = 4.80 µM) being the most effective growth inhibitor among the three. As for compounds **20** and **22**, both were found to possess rather similar growth-inhibitory properties (GI_50_ = 8.00 µM and 7.40 µM, respectively). The remaining labdane diterpenes were reported to moderately inhibit the growth of the A-549 cell line in the following order: **24** > **18** > **23** > **19 [36]**.

##### NCI-H187 Cell Line

Songsri and Nuntawong in their investigation revealed that the NCI-H187 cell line in comparison to the MCF-7 and KB cell lines was more susceptible to the cytotoxic activity of the labdane-type diterpenes which they had isolated and characterized from the hexane and dichloromethane extracts of the rhizomes of *H. ellipticum* [39]. Among the ten labdane-type diterpenes, the NCI-H187 cells were resistant to only two of them, zerumin A and (*E*)-14,15,16-trinorlabda-8(17),11-dien-13-oic acid. The IC_50_ values for both these compounds were above 100 µM. Compound **29** (IC_50_ = 0.40 µM) was regarded as potent. Its cytotoxic activity was comparable to doxorubicin (IC_50_ = 0.10 µM) but found to be four and a half-folds stronger than that of ellipticine (IC_50_ = 1.79 µM). Compounds **16** (IC_50_ = 2.18 µM) and **30** (IC_50_ = 2.72 µM) were almost equally cytotoxic toward the NCI-H187 cells. Both labdane diterpenes were considered to be comparable to ellipticine (IC_50_ = 1.79 µM) in terms of their cytotoxicity [39]. The remaining diterpenes were either moderately (**6**) or weakly (**20**, **27**, **28**, **31**) cytotoxic. The rather weak cytotoxicity of compounds **20**, **27**, **28**, and **31** upon comparison to compounds **6**, **16**, **29**, and **30** implied that the presence of an α,β-unsaturated γ-lactone ring in the side chain played an essential role in facilitating the cytotoxic activity against the tested cell line. When the cytotoxicity of compounds **16**, **29**, and **30** was compared to that of compound **6**, the greater activity of compounds **16**, **29**, and **30** led to the assumption that the potency of these three compounds could have resulted from the presence of a *trans* double bond which was conjugated to the α,β-unsaturated γ-lactone ring in their respective side chains in contrast to compound **6** in which the *trans* double bond was absent. The variation in the α,β-unsaturated γ-lactone rings of compounds **16**, **29** and **30** could further explain the significant difference in the cytotoxicities between these labdane diterpenes. The substitution of position C-16 of the α,β-unsaturated γ-lactone ring in compound **16** with a hydroxyl group, as well as the substitution of position C-15 of the α,β-unsaturated γ-lactone ring in compound **30** with a methoxyl group, has lowered their cytotoxicities by almost six- to seven-fold compared to compound **29**, in which none of its carbon atoms in its α,β-unsaturated γ-lactone ring were substituted with an oxygenated functional group.

In 2010, Kumrit et al. screened the hexane and dichloromethane extracts prepared from the rhizomes of *H. gardnerianum* for their cytotoxicity against the NCI-H187 cell line. Preliminary results of their REMA assay revealed that both extracts exhibited significant cytotoxicity [41]. The extracts were subsequently purified, and the isolates were characterized in order to identify the secondary metabolites which could have been responsible for the respective extract’s cytotoxicity. A total of seven cytotoxic labdane diterpenes (**14**, **27**, **29**, **32**, **33**, **34**, and **35**) were identified. The most cytotoxic labdane diterpene was compound **29** (IC_50_ = 0.40 µM) followed by compounds **35** (IC_50_ = 2.46 µM) and **34** (IC_50_ = 3.10 µM). Interestingly, the cytotoxicity of compound **29** was almost 4.5-fold more potent than that of ellipticine (1.79uM). The remaining compounds (**14**, **27**, **32**, and **33**) only weakly inhibited the cytotoxic activity of the NCI-H187 cells [41]. It was evident that although compounds **14**, **27**, and **32** had a *trans* double bond conjugated to a furan ring in their respective side chains, the presence of a hydroxyl group bonded to either position C-6 or C-7 of the decalin moieties of compounds **14** and **32** tends to have slightly increased the activity of these compounds compared to that of compound **27** in which its decalin moiety does not bear a hydroxyl group at the corresponding positions. With regards to the activity of compounds **29** and **33–35**, although all four compounds have a *trans* double bond which is conjugated to an α,β-unsaturated γ-lactone ring in their respective side chains, the variation in the α,β-unsaturated γ-lactone rings in addition to the variation in the decalin moieties could have contributed to the differences in the extent of their cytotoxic activities. For example, the presence of a hydroxyl group at positions C-6 and C-7 in the respective decalin moieties of compounds **33** and **34** resulted in a significant decrease in their activities in contrast to compound **29** whose decalin moiety does not bear a hydroxyl group at either one of the corresponding positions. Similarly, when the activities of compounds **34** and **35** were compared to one another, although both compounds bore a hydroxyl group at position C-7 of their respective decalin moieties, the presence of a hydroxyl group bonded to the α,β-unsaturated γ-lactone ring in compound **35** slightly increased its activity compared to that of compound **34** whose α,β-unsaturated γ-lactone ring does not bear a hydroxyl group.

#### 4.2.7. Prostate Cancer Cell Lines

DU145 is the only prostate cancer cell line reported in a study included in this review to evaluate the cytotoxicity of the labdane-type diterpenes isolated from the Zingiberaceae (Table 20). Till present, there has been only a single report on the cytotoxic potential of either labdane diterpenes, norlabdane diterpenes, or bis-labdanic diterpenes towards this particular prostate cancer cell line. Compound **6**, isolated and characterized from the rhizomes of *C. manga,* exhibited a moderate cytotoxicity against the DU145 cell line, and its IC_50_ value was measured to be 11.21 µM [33]. Compound **6** was once again, like in the cases of the MCF-7 and HepG2 cell lines, found to be more cytotoxic compared to the curcuminoids isolated alongside it. Demethoxycurcumin (IC_50_ = 21.70 µM), bisdemethoxycurcumin (IC_50_ = 30.10 µM), and curcumin (IC_50_ = 20.60 µM) were less effective in inhibiting the cytotoxic activity of the DU145 cells compared to compound **6 [33]**. The reported IC_50_ value of compound **6** was lower than the compound’s IC_50_ value against the HepG2 liver cancer cell line (IC_50_ = 25.33 µM), but higher than the compound’s IC_50_ value against the MCF-7 breast cancer cell line (IC_50_ = 0.59 µM), within the same study [33]. Therefore, we could conclude that compound **6** is the most cytotoxic towards the MCF-7 breast cancer cells, followed by the DU145 prostate cancer cells and then finally the HepG2 liver cancer cells [33].

## 5. Strengths and Limitations

The results in this review were summarized from 17 studies that reported on the cytotoxicity of the labdane diterpenes, norlabdane diterpenes, and bis-labdanic diterpenes which were isolated and characterized from the Zingiberaceae against various types of cancer cell lines. This systematic review provides a comprehensive overview of the relevant studies from 2000 to 2022. This review adheres to the Preferred Reporting Items for Systematic Reviews and Meta-Analyses (PRISMA).

In regards to the limitations, the studies included in this review are limited to the in vitro cytotoxic studies against the various types of cancer cell lines. Furthermore, the types of cancer cell lines included in the review are also limited and do not include every type of cancer. The several types of cancer cell lines included in this review have a very limited number of studies carried out on them. There is also a lack of consistency in the reporting of the cytotoxicities by the studies included. The cytotoxicities of the labdane diterpenes, norlabdane diterpenes, and bis-labdanic diterpenes were reported in both half-maximal inhibitory concentration and half-maximal growth inhibition. Furthermore, the studies included in this review applied different assays to measure the cytotoxic activities of the labdane-type diterpenes. The positive controls which were tested alongside the labdane-type diterpenes in the different studies were also different. Certain studies do not include the IC_50_ values for the positive control. Therefore, comparing the effectiveness of the labdane-type diterpenes in inhibiting the cytotoxic activity of a given cell line to the positive control between different studies cannot be carried out conclusively.

As a systematic review, this review only serves to provide an overview of the existing literature and does not aim to assess the quality of the studies.

## 6. Future Research

Due to the various side effects of therapies currently used in the treatment of cancer, there continues to be a need for the search for alternatives. Several types of cancers, such as pancreatic, cervical, and prostate cancers, have little to no studies conducted to evaluate the cytotoxicities of the labdane diterpenes, norlabdane diterpenes, and bis-labdanic diterpenes from the Zingiberaceae. Future studies should focus on the effects of the labdane diterpenes, norlabdane diterpenes, and bislabdanic diterpenes against these cancer types that currently have fewer studies conducted, and therefore fewer data points to consider. There is also a lack of studies on the mechanism of action of the cytotoxicities against the tested cell lines. Further studies of the cytotoxicities against cancer cells should also investigate the mechanism of action in addition to the half-maximal inhibitory concentration and half-maximal growth inhibition of the labdane diterpenes, norlabdane diterpenes, and bis-labdanic diterpenes.

## 7. Conclusions

In the present review, forty-four labdane-type diterpenes from five different genera of the Zingiberaceae, namely *Alpinia*, *Amomum*, *Curcuma*, *Hedychium* and *Roscoea* which exhibited cytotoxicity with IC_50_ < 100 µM were discussed. The subclasses of the labdane-type diterpenes which demonstrated cytotoxic activity were the labdane diterpenes, norlabdane diterpenes, and bis-labdanic diterpenes. The genus *Hedychium* was the richest source of active compounds.

The cytotoxicity of the labdane-type diterpenes was established on various types of cancer cell lines, in particular the most common types of cancers such as breast cancer (MCF-7, T-47D, and MDA-MB-231 cell lines), cervical cancer (KB, SMMC-7721, SGC-7901, and HeLa cell lines), liver cancer (HepG2 and HCC-S102 cell lines), colorectal cancer (HCT-116, HT-29, COLO 205 and DLD-1 cell lines), pancreatic cancer (BxPC-3 cell line), lung cancer (A-549 and NCI-H187 cell lines), and prostate cancer (DU145 cell line).

Activity with IC_50_ < 10 µM was obtained with two compounds (**6** and **16**) against the MCF-7 cell line, one compound (**17**) against the T-47D cell line, three compounds (**7**, **17**, and **20**) against the MDA-MB-231 cell line, five compounds (**1**, **2**, **16**, **17** and **20**) against the KB cell line, two compounds (**17** and **25**) against the HeLa cell line, three compounds (**15**, **16** and **17**) against the HepG2 cell line, one compound (**7**) against the HCT-116 cell line, four compounds (**10**, **11**, **12** and **17**) against the HT-29 cell line, four compounds (**20**, **21**, **22** and **33**) against the A549 cell line, and five compounds (**16**, **29**, **30**, **34** and **35**) against the NCI-H187 cell line. The presence of either a γ-lactone ring or an α,β-unsaturated γ-lactone ring in the side chain of a majority of these labdane-type diterpenes could have contributed to their potencies. Among the abovementioned nineteen labdane-type diterpenes reported to have IC_50_ values lower than 10 µM, only compounds **16** and **17** were found to be active across several types of cell lines. Compound **16** actively inhibited the cytotoxic activities of four types of cell lines which include the MCF-7, KB, HepG,2, and NCI-H187 cell lines. Compound **17** on the other hand was effective in inhibiting the activities of the T-47D, MDA-MB-231, KB, HepG2, and HT-29 cell lines.

The present review thus provides an insight into the potential of the Zingiberaceae as a source of natural anticancer drugs bearing the labdane skeleton.

## Figures and Tables

**Table 1:** Keywords used for search strategy.

	Keywords
Bioactive compounds	“Labdane diterpene” OR “Labdane diterpenes” OR “Labdane diterpenoid” OR “Labdane diterpenoids” OR “Bis-labdanic diterpene” OR “Bis-labdanic diterpenes” OR “Bis-labdanic diterpenoid” OR “Bis-labdanic diterpenoids” OR “Dimeric labdane diterpene” OR “Dimeric labdane diterpenes” OR “Dimeric labdane diterpenoid” OR “Dimeric labdane diterpenoids”
Cytotoxic	“Cytotoxic”

**Table 2:** Inclusion and exclusion criteria.

Inclusion Criteria	Exclusion Criteria
Population: in vitro studies	Studies with incomplete results/methodologies; in vivo and ex vivo studies
Intervention: naturally occurring labdane-type diterpenes from the Zingiberaceae	Synthetic labdane-type diterpenes
Comparators: standard (synthetic or natural) compounds	Articles that do not have comparators
Outcome: articles that report IC_50_ or GI_50_ values of the cytotoxic activities of the labdane-type diterpenes	Articles that do not report the IC_50_ or GI_50_ values of the cytotoxic activities of the labdane-type diterpenes
Articles in English	Articles not in English
Years: 2000–March 2022	Human trials, clinical trials, randomized control trials, animal studies, literature reviews, systemic reviews, meta-analyses, conference proceedings, and patents
Original articles including short communications, notes and letters	Studies without accessible full text

**Table 3:** Cytotoxic labdane-type diterpenes from the Zingiberaceae.

Species	Name of Labdane-Type Diterpenes	Reference
*Alpinia calcarata* Rosc.	Calcaratarin D (**1**) Calcaratarin E (**2**)	[30]
*Alpinia intermedia* Gagnep	Intermedin A (**3**)	[31]
*Amomum maximum* Roxb	Amomax C (**4**) (12*Z*,14*R*)-labda-8(17),12- diene-14,15,16-triol (**5**)	[32]
*Curcuma mangga* Val.van Zip	Zerumin B (**6**)	[33]
*Curcuma mutabilis* Škorničk., M. Sabu & Prasanthk.	(*E*)-14,15-epoxylabda-8(17),12-dien-16-al (Cm epoxide) (**7**)	[34]
*Hedychium coronarium* J. Koenig	Hedycoronal A (**8**) Hedycoronal B (**9**) Coronarin D methyl ether (**10**) Coronarin D ethyl ether (**11**) Labda-8(17),11,13-trien-15(16)-olide (**12**) (12*E*)-Labda-8(17),12-dien-15(16)-olide (**13**) Coronarin A (**14**) 15-Hydroxy-11,15-epoxylabda-8(17),12-dien-16-al (**15**) 16-Hydroxylabda-8(17),11,13-trien-15,16-olide (**16**) Isocoronarin D (**17**)	[35]
*Hedychium coronarium* J. Koenig	Coronarin D methyl ether (**10**) 6-oxo-7,11,13-labdatrien-17-al-16,15-olide (**18**) 7,17-dihydroxy-6-oxo-7,11,13-labdatrien-16,15-olide (**19**) Coronarin D (**20**) Coronarin C (**21**) Hedychenone (**22**) 6-oxo-7,11,13-labdatriene-16,15-olide (**23**) Pacovatinin A (**24**)	[36]
*Hedychium coronarium* J. Koenig	Isocoronarin D (**17**) Coronarin D (**20**) Coronarin B (**25**)	[37]
*Hedychium coronarium* J. Koenig	Coronarin A (**14**) Calcaratarin A (**26**)	[38]
*Hedychium ellipticum* Buch-Ham. ex Sm.	Zerumin B (**6**) 16-Hydroxylabda-8(17),11,13-trien-15,16-olide (**16**) Coronarin D (**20**) Coronarin E (**27**) (*E*)-15,16-Bisnorlabda-8(17),11-dien-13-one (**28**) Villosin (**29**) 15-Methoxylabda-8(17),11,13-trien-15,16-olide (**30**) (*E*)-labda-8(17),12-dien-15,16-dial (**31**)	[39]
*Hedychium forrestii* Tong.	Yunnancoronarin A (**32**) Yunnancoronarin B (**33**)	[40]
*Hedychium gardnerianum* Sheppard ex Ker Gawl	Coronarin A (**14**) Coronarin E (**27**) Villosin (**29**) Yunnancoronarin A (**32**) Yunnancoronarin B (**33**) Hedyforrestin B (**34**) Hedyforrestin C (**35**)	[41]
*Hedychium longipetalum* X. Hu & N. Liu	Yunnancoronarin A (**32**) Hedyforrestin B (**34**) Hedyforrestin C (**35**) Hedylongnoid C (**36**)	[42]
*Hedychium spicatum* Buch.-Ham. ex Sm.	Coronarin E (**27**) 7-hydroxy,15,16-epoxy-17-al-7,11,13(16),14-labda-tetraene-6-one (7-hydroxy hedichinal) (**37**) 14,15,16-trinor-7,11-labdadien-13-oicacid (spicatanoic acid) (**38**) Yunnancoronarin D (**39**)	[43]
*Hedychium spicatum* Buch.-Ham. ex Sm.	Yunnancoronarin A (**32**) 7-hydroxy,6-oxo-7,11,13-labdatrien-16,15-olide (hedychilactone D) (**40**) 9-hydroxy,15,16-epoxy-7,11,13(16)14-labdatetraen-6-one (9-hydroxy hedychenone) (**41**)	[44]
*Hedychium yunnanense* Gagnep.	Hedychenone (**22**) Villosin (**29**) Hedychenoid B (**42**)	[45]
*Roscoea purpurea* Sm.	Coronarin A (**14**) Coronarin K (**43**) Coronarin L (**44**)	[46]

**Table 4:** Cytotoxic activities of labdane-type diterpenes from the Zingiberaceae against MCF-7 breast cancer cell line.

Compound	IC_50_/GI_50_ (µM)		Assay	Reference
	MCF-7	Potency		
Amomax C **(4)** 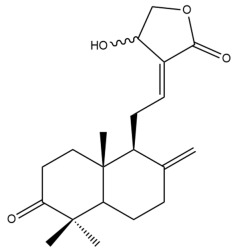	IC_50_: 29.80 ± 2.41	Moderate	MTT	[32]
Zerumin B **(6)** 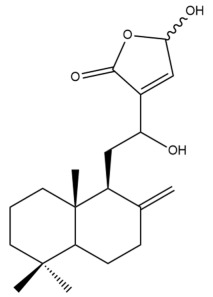	IC_50_: 0.59 ± 1.8	**Potent**	MTT	[33]
	IC_50_: 39.71	Weak	REMA	[39]
Coronarin D methyl ether **(10)** 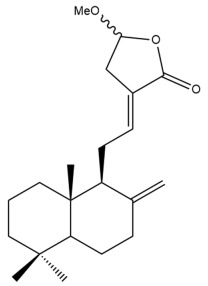	GI_50_: 25.3 ± 2.8	Moderate	SRB	[36]
Coronarin A **(14)** 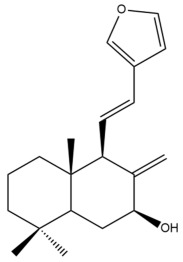	IC_50_: >50	Weak	MTT	[46]
16-Hydroxylabda-8(17),11,13-trien-15,16-olide **(16)** 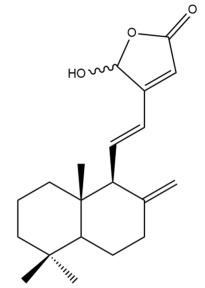	IC_50_: 8.75	**Strong**	REMA	[39]
6-oxo-7,11,13-labdatrien-17-al-16,15-olide **(18)** 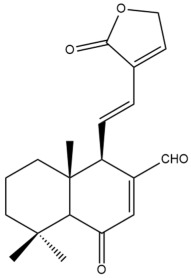	GI_50_: 69.9 ± 8.5	Weak	SRB	[36]
7,17-dihydroxy-6-oxo-7,11,13-labdatrien-16,15-olide **(19)** 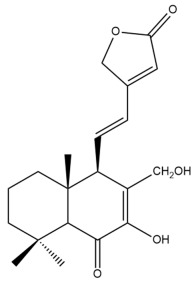	GI_50_: 12.0 ± 1.4	Moderate	SRB	[36]
Coronarin D **(20)** 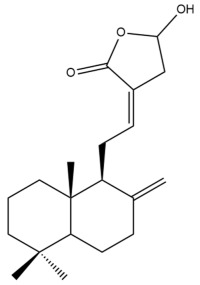	GI_50_: 17.4 ± 2.1	Moderate	SRB	[36]
	IC_50_: 51.03	Weak	REMA	[39]
Coronarin C **(21)** 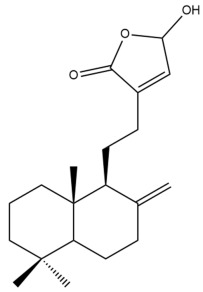	GI_50_: 32.6 ± 3.6	Weak	SRB	[36]
Hedychenone **(22)** 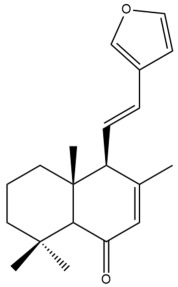	GI_50_: 14.9 ± 2.3	Moderate	SRB	[36]
6-oxo-7,11,13-labdatriene-16,15-olide **(23)** 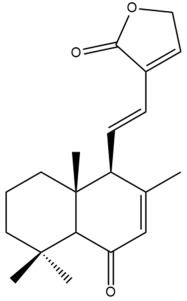	GI_50_: 15.7 ± 2.4	Moderate	SRB	[36]
Pacovatinin A **(24)** 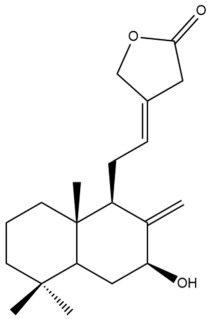	GI_50_: 44.7 ± 5.3	Weak	SRB	[36]
Villosin **(29)** 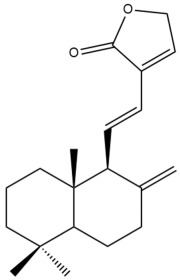	IC_50_: 28.29	Moderate	REMA	[39]
(*E*)-labda-8(17),12-dien-15,16-dial **(31)** 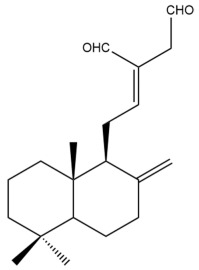	IC_50_: 74.56	Weak	REMA	[39]
Yunnancoronarin A **(32)** 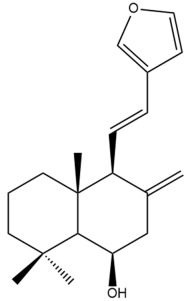	IC_50_: 93.5 ± 0.27	Weak	NA	[44]
7-hydroxy,6-oxo-7,11,13-labdatrien-16,15-olide (hedychilactone D) **(40)** 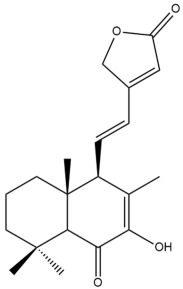	IC_50_: 63.76 ± 0.12	Weak	NA	[44]
9-hydroxy,15,16-epoxy-7,11,13(16)14-labdatetraen-6-one (9-hydroxy hedychenone) **(41)** 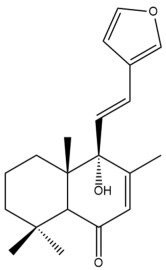	IC_50_: 98.50 ± 0.19	Weak	NA	[44]
Coronarin K **(43)** 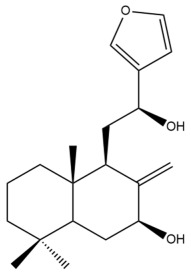	IC_50_: 56.24 ± 0.83	Weak	MTT	[46]
Coronarin L **(44)** 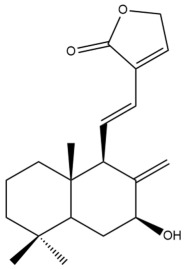	IC_50_: 49.84 ± 2.61	Weak	MTT	[46]

**Table 5:** Cytotoxic activities of labdane-type diterpenes from the Zingiberaceae against T-47D breast cancer cell line.

Compound	IC_50_/GI_50_ (µM)		Assay	Reference
	T-47D	Potency		
Isocoronarin D **(17)** 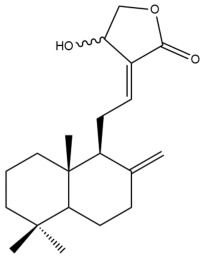	IC_50_: 8.49	**Strong**	MTT	[37]
Coronarin D **(20)** 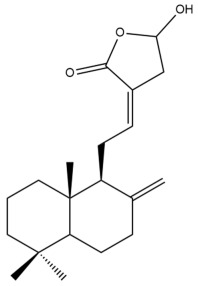	IC_50_: 15.08	Moderate	MTT	[37]

**Table 6:** Cytotoxic activities of labdane-type diterpenes from the Zingiberaceae against MDA-MB-231 breast cancer cell line.

Compound	IC_50_/GI_50_ (µM)		Assay	Reference
	MDA-MB-231	Potency		
Isocoronarin D **(17)** 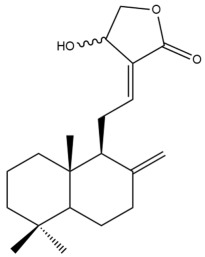	IC_50_: 7.23	**Strong**	MTT	[37]
Coronarin D **(20)** 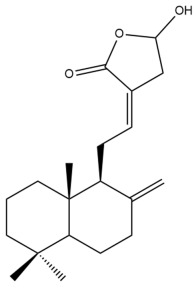	IC_50_: 7.86	**Strong**	MTT	[37]

**Table 7:** Cytotoxic activities of labdane-type diterpenes from the Zingiberaceae against KB cervical cancer cell line.

Compound	IC_50_/GI_50_ (µM)		Assay	Reference
	KB	Potency		
Calcaratarin D **(1)** 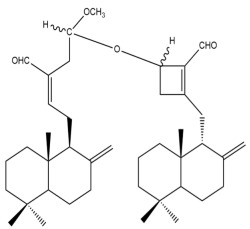	IC_50_: 0.34	**Potent**	MTT	[30]
Calcaratarin E **(2)** 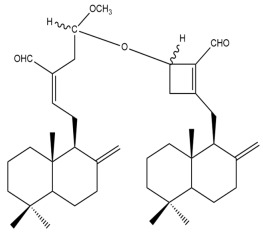	IC_50_: 0.24	**Potent**	MTT	[30]
Zerumin B **(6)** 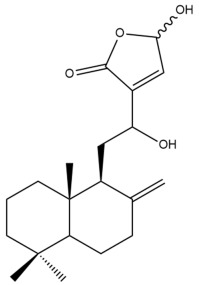	IC_50_: 39.26	Weak	REMA	[39]
16-Hydroxylabda-8(17),11,13-trien-15,16-olide **(16)** 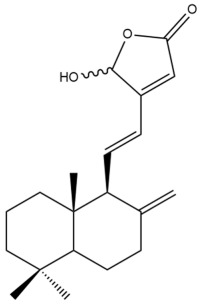	IC_50_: 2.75	**Strong**	REMA	[39]
Isocoronarin D **(17)** 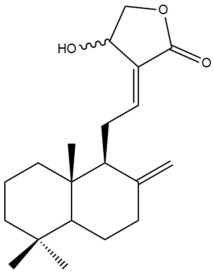	IC_50_: 8.81	**Strong**	MTT	[37]
Coronarin D **(20)** 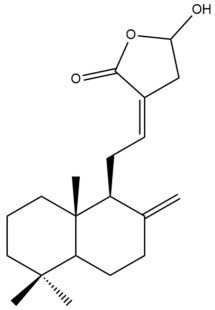	IC_50_: 9.43	**Strong**	MTT	[37]
Coronarin E **(27)** 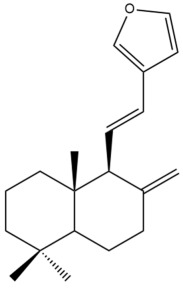	IC_50_: 34.00	Weak	REMA	[39]
Villosin **(29)** 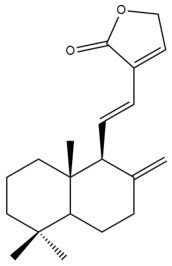	IC_50_: 13.65	Moderate	REMA	[39]
15-Methoxylabda-8(17),11,13-trien-15,16-olide **(30)** 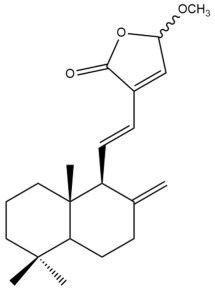	IC_50_: 72.08	Weak	REMA	[39]
(*E*)-labda-8(17),12-dien-15,16-dial **(31)** 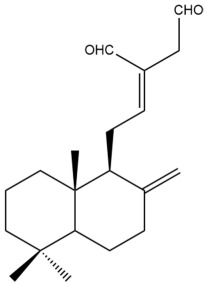	IC_50_: 96.05	Weak	REMA	[39]

**Table 8:** Cytotoxic activities of labdane-type diterpenes from the Zingiberaceae against SMMC-7721 cervical cancer cell line.

Compound	IC_50_/GI_50_ (µM)		Assay	Reference
	SMMC-7721	Potency		
Amomax C **(4)** 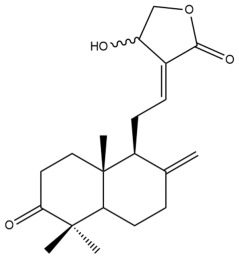	IC_50_: 24.38 ± 1.81	Moderate	MTT	[32]

**Table 9:** Cytotoxic activities of labdane-type diterpenes from the Zingiberaceae against SGC-7901 cervical cancer cell line.

Compound	IC_50_/GI_50_ (µM)		Assay	Reference
	SGC-7901	Potency		
Hedychenone **(22)** 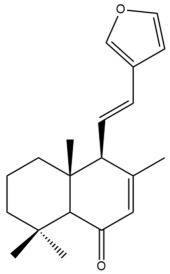	IC_50_: 23.73 ± 0.7	Moderate	SRB	[45]
Villosin **(29)** 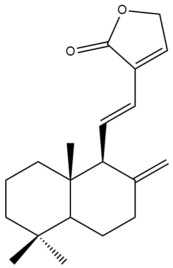	IC_50_: 25.87 ± 0.7	Moderate	SRB	[45]
Yunnancoronarin A **(32)** 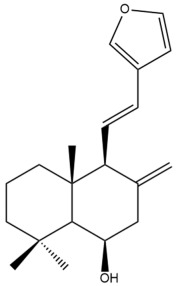	IC_50_: 20.7	Moderate	SRB	[42]
Hedyforrestin B **(34)** 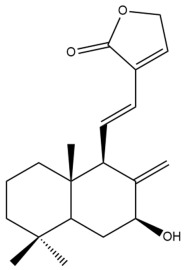	IC_50_: 45.98	Weak	SRB	[42]
Hedyforrestin C **(35)** 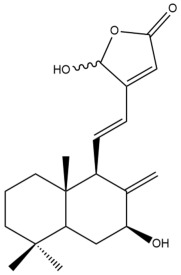	IC_50_: 21.96	Moderate	SRB	[42]
Hedylongnoid C **(36)** 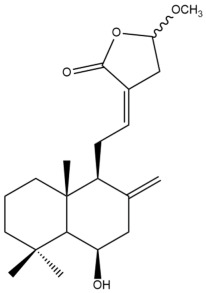	IC_50_: 25.1	Moderate	SRB	[42]
Hedychenoid B **(42)** 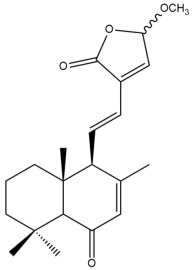	IC_50_: 42.98 ± 1.5	Weak	SRB	[45]

**Table 10:** Cytotoxic activities of labdane-type diterpenes from the Zingiberaceae against HeLa cervical cancer cell line.

Compound	IC_50_/GI_50_ (µM)		Assay	Reference
	HeLa	Potency		
Hedycoronal A **(8)** 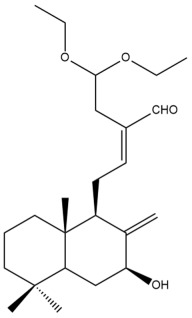	IC_50_: 37.0 ± 0.4	Weak	SRB	[35]
Coronarin D methyl ether **(10)** 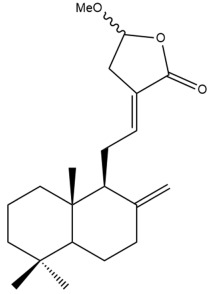	IC_50_: 88.1 ± 0.6	Weak	SRB	[35]
(12*E*)-Labda-8(17),12-dien-15(16)-olide **(13)** 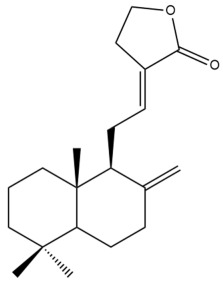	IC_50_: 33.0 ± 0.5	Weak	SRB	[35]
Coronarin A **(14)** 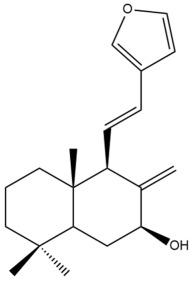	IC_50_: 45.2 ± 0.3	Weak	SRB	[35]
15-Hydroxy-11,15-epoxylabda-8(17),12-dien-16-al **(15)** 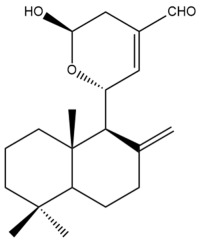	IC_50_: 28.7 ± 0.4	Moderate	SRB	[35]
16-Hydroxylabda-8(17),11,13-trien-15,16-olide **(16)** 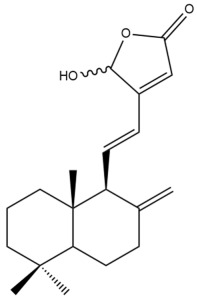	IC_50_: 23.0 ± 0.4	Moderate	SRB	[35]
Isocoronarin D **(17)** 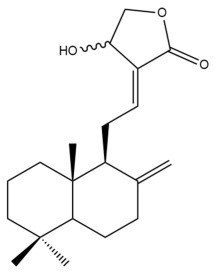	IC_50_: 9.12	**Strong**	MTT	[37]
6-oxo-7,11,13-labdatrien-17-al-16,15-olide **(18)** 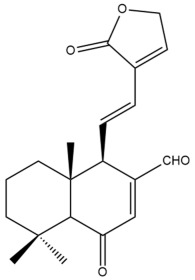	GI_50_: 76.9 ± 6.2	Weak	SRB	[36]
7,17-dihydroxy-6-oxo-7,11,13-labdatrien-16,15-olide **(19)** 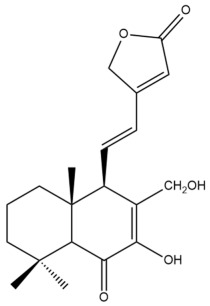	GI_50_: 79.7 ± 5.8	Weak	SRB	[36]
Coronarin D **(20)** 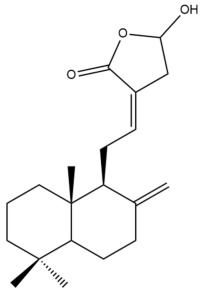	IC_50_: 12.57	Moderate	MTT	[37]
	GI_50_: 47.0 ± 5.6	Weak	SRB	[36]
Coronarin C **(21)** 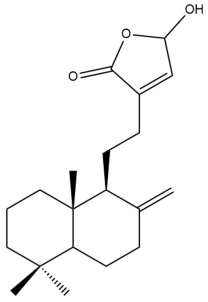	GI_50_: 36.3 ± 4.3	Weak	SRB	[36]
Hedychenone **(22)** 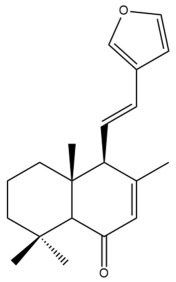	GI_50_: 63.2 ± 5.4	Weak	SRB	[36]
	IC_50_: 32.71 ± 1.61	Weak	SRB	[45]
6-oxo-7,11,13-labdatriene-16,15-olide **(23)** 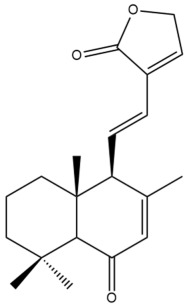	GI_50_: 83.6 ± 6.7	Weak	SRB	[36]
Pacovatinin A **(24)** 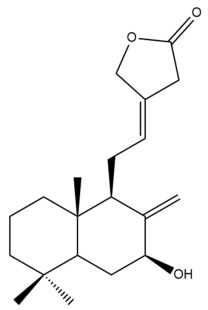	GI_50_: 62.1 ± 4.7	Weak	SRB	[36]
Coronarin B **(25)** 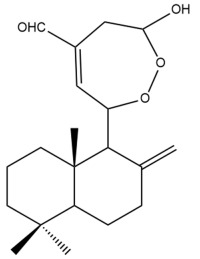	IC_50_: 8.54	**Strong**	MTT	[37]
Villosin **(29)** 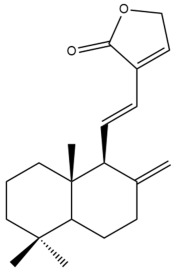	IC_50_: 44.13 ± 2.1	Weak	SRB	[45]
Yunnancoronarin A **(32)** 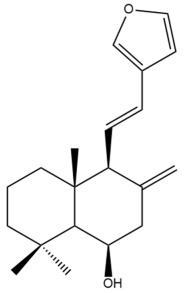	IC_50_: 21.93	Moderate	SRB	[42]
Hedyforrestin B **(34)** 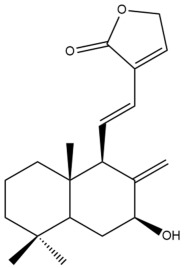	IC_50_: 46.93	Weak	SRB	[42]
Hedyforrestin C **(35)** 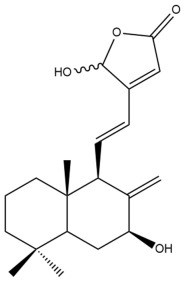	IC_50_: 27.74	Moderate	SRB	[42]
Hedylongnoid C **(36)** 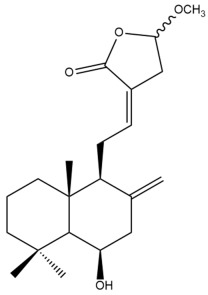	IC_50_: 32.48	Weak	SRB	[42]

**Table 11:** Cytotoxic activities of labdane-type diterpenes from the Zingiberaceae against HepG2 liver cancer cell line.

Compound	IC_50_ (µM)		Assay	Reference
	HepG2	Potency		
Intermedin A **(3)** 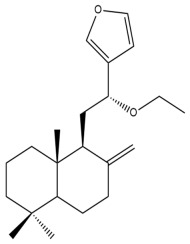	IC_50_: 119.82 ± 2.37	Weak	MTT	[31]
(12*Z*,14*R*)-labda-8(17),12- diene-14,15,16-triol **(5)** 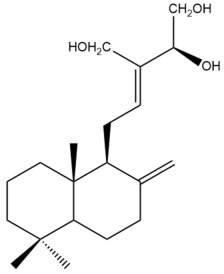	IC_50_: 30.1 ± 1.86	Weak	MTT	[32]
Zerumin B **(6)** 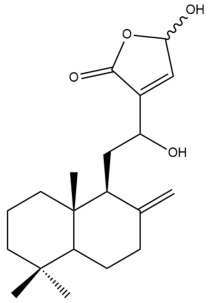	IC_50_: 25.33 ± 3.3	Moderate	MTT	[33]
Hedycoronal A **(8)** 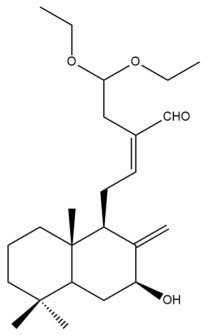	IC_50_: 15.6 ± 0.6	Moderate	SRB	[35]
Hedycoronal B **(9)** 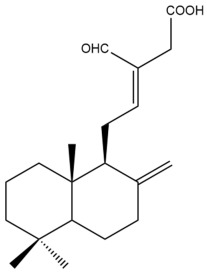	IC_50_: 26.7 ± 0.4	Moderate	SRB	[35]
Coronarin D methyl ether **(10)** 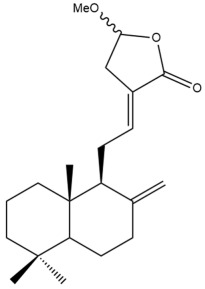	IC_50_: 14.2 ± 0.3	Moderate	SRB	[35]
Coronarin D ethyl ether **(11)** 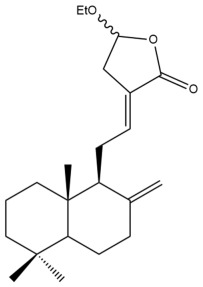	IC_50_: 13.4 ± 0.2	Moderate	SRB	[35]
Labda-8(17),11,13-trien-15(16)-olide **(12)** 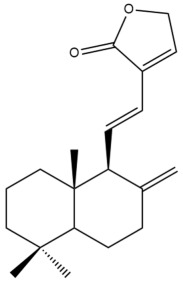	IC_50_: 12.6 ± 0.1	Moderate	SRB	[35]
(12*E*)-Labda-8(17),12-dien-15(16)-olide **(13)** 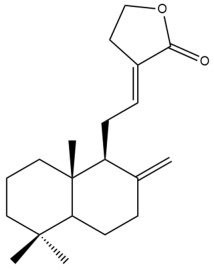	IC_50_: 10.6 ± 0.4	Moderate	SRB	[35]
Coronarin A **(14)** 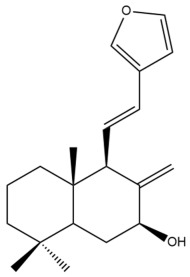	IC_50_: 10.7 ± 0.4	Moderate	SRB	[35]
15-Hydroxy-11,15-epoxylabda-8(17),12-dien-16-al **(15)** 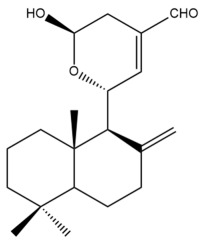	IC_50_: 0.9 ± 0.2	**Potent**	SRB	[35]
16-Hydroxylabda-8(17),11,13-trien-15,16-olide **(16)** 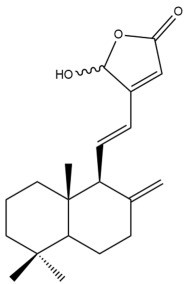	IC_50_: 1.1 ± 0.2	**Strong**	SRB	[35]
Isocoronarin D **(17)** 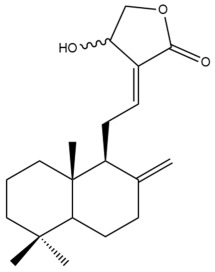	IC_50_: 8.0 ± 0.5	**Strong**	SRB	[35]
	IC_50_: 16.67	Moderate	MTT	[37]
Coronarin D **(20)** 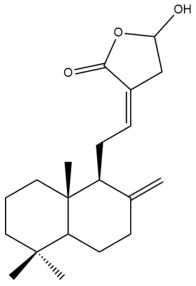	IC_50_: 56.56	Weak	MTT	[37]
Coronarin B **(25)** 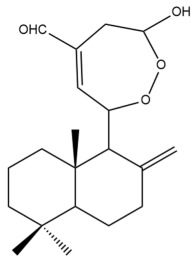	IC_50_: 24.51	Moderate	MTT	[37]

**Table 12:** Cytotoxic activities of labdane-type diterpenes from the Zingiberaceae against HCC-S102 liver cancer cell line.

Compound	IC_50_ (µM)		Assay	Reference
	HCC-S102	Potency		
Isocoronarin D **(17)** 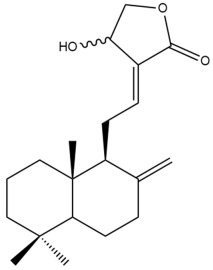	IC_50_: 36.16	Weak	MTT	[37]
Coronarin D **(20)** 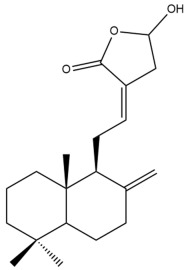	IC_50_: 67.56	Weak	MTT	[37]

**Table 13:** Cytotoxic activities of labdane-type diterpenes from the Zingiberaceae against HCT-116 colorectal cancer cell line.

Compound	IC_50_ (µM)		Assay	Reference
	HCT-116	Potency		
(*E*)-14,15-epoxylabda-8(17),12-dien-16-al (Cm epoxide) **(7)** 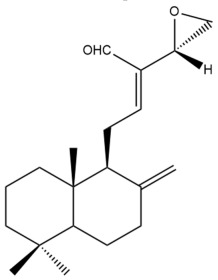	IC_50_: 3.21	**Strong**	MTT	[34]
Coronarin A **(14)** 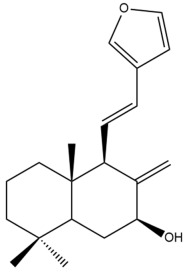	IC_50_: >50	Weak	MTT	[46]
Coronarin K **(43)** 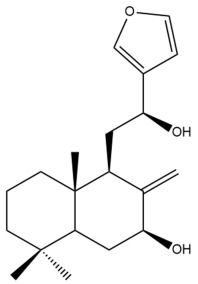	IC_50_: 26.03 ± 1.46	Moderate	MTT	[46]
Coronarin L **(44)** 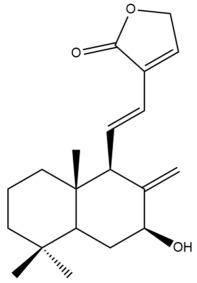	IC_50_: >50	Weak	MTT	[46]

**Table 14:** Cytotoxic activities of labdane-type diterpenes from the Zingiberaceae against HT-29 colorectal cancer cell line.

Compound	IC_50_ (µM)		Assay	Reference
	HT-29	Potency		
Hedycoronal A **(8)** 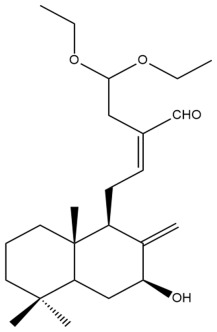	IC_50_: 18.7 ± 0.4	Moderate	SRB	[35]
Hedycoronal B **(9)** 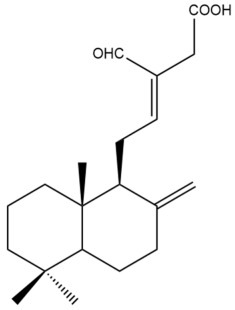	IC_50_: 16.2 ± 0.2	Moderate	SRB	[35]
Coronarin D methyl ether **(10)** 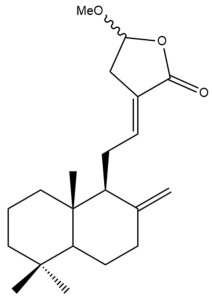	IC_50_: 7.8 ± 0.2	**Strong**	SRB	[35]
Coronarin D ethyl ether **(11)** 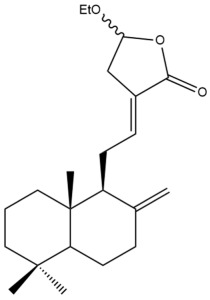	IC_50_: 9.8 ± 0.4	**Strong**	SRB	[35]
Labda-8(17),11,13-trien-15(16)-olide **(12)** 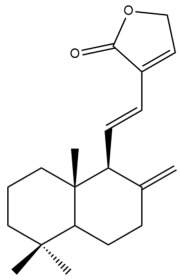	IC_50_: 7.8 ± 0.3	**Strong**	SRB	[35]
(12*E*)-Labda-8(17),12-dien-15(16)-olide **(13)** 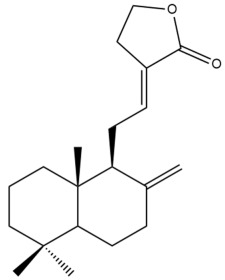	IC_50_: 13.9 ± 0.1	Moderate	SRB	[35]
Coronarin A **(14)** 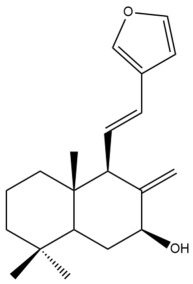	IC_50_: 14.7 ± 0.6	Moderate	SRB	[35]
15-Hydroxy-11,15-epoxylabda-8(17),12-dien-16-al **(15)** 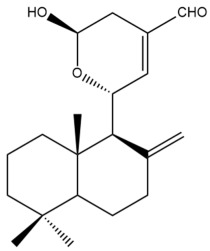	IC_50_: 15.6 ± 0.6	Moderate	SRB	[35]
16-Hydroxylabda-8(17),11,13-trien-15,16-olide **(16)** 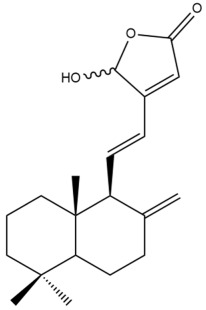	IC_50_: 12.3 ± 0.3	Moderate	SRB	[35]
Isocoronarin D **(17)** 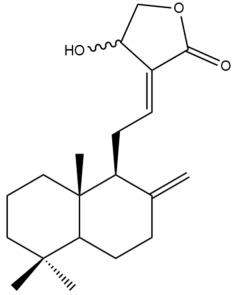	IC_50_: 8.8 ± 0.1	**Strong**	SRB	[35]

**Table 15:** Cytotoxic activities of labdane-type diterpenes from the Zingiberaceae against COLO 205 colorectal cancer cell line.

Compound	IC_50_ (µM)		Assay	Reference
	COLO 205	Potency		
Yunnancoronarin A **(32)** 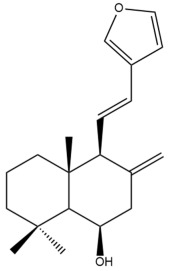	IC_50_: 90.47 ± 0.1	Weak	NA	[44]
7-hydroxy,6-oxo-7,11,13-labdatrien-16,15-olide (hedychilactone D) **(40)** 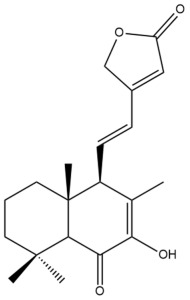	IC_50_: 36.44 ± 0.09	Weak	NA	[44]
9-hydroxy,15,16-epoxy-7,11,13(16)14-labdatetraen-6-one (9-hydroxy hedychenone) **(41)** 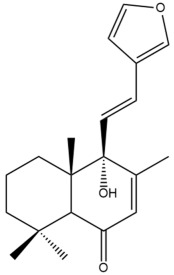	IC_50_: 76.47 ± 0.03	Weak	NA	[44]

**Table 16:** Cytotoxic activities of labdane-type diterpenes from the Zingiberaceae against DLD-1 colorectal cancer cell line.

Compound	IC_50_ (µM)		Assay	Reference
	DLD-1	Potency		
Coronarin A **(14)** 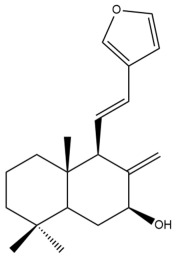	IC_50_: 41.67 ± 6.33	Weak	MTT	[38]
Calcaratarin A **(26)** 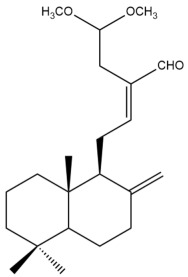	IC_50_: 35.34 ± 6.61	Weak	MTT	[38]

**Table 17:** Cytotoxic activities of labdane-type diterpenes from the Zingiberaceae against selected pancreatic cancer cell lines.

Compound	IC_50_ (µM)		Assay	Reference
	Bxpc-3	Potency		
Coronarin A (**14**) 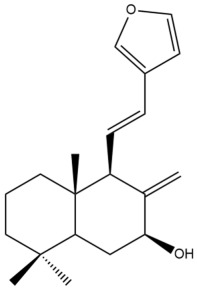	IC_50_: 22.83 ± 1.47	Moderate	MTT	[46]
Coronarin K (**43**) 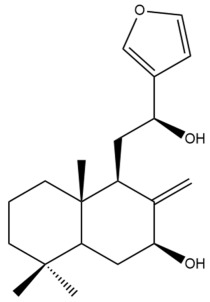	IC_50_: 56.70 ± 2.17	Weak	MTT	[46]
Coronarin L (**44**) 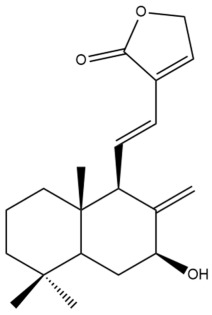	IC_50_: 56.83 ± 1.92	Weak	MTT	[46]

**Table 18:** Cytotoxic activities of labdane-type diterpenes from the Zingiberaceae against A-549 lung cancer cell line.

Compound	IC_50_/GI_50_ (µM)		Assay	Reference
	A-549	Potency		
Coronarin D methyl ether **(10)** 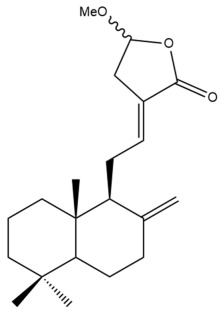	GI_50_: 30 ± 3.9	Moderate	SRB	[36]
Coronarin A **(14)** 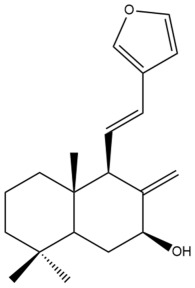	IC_50_: 61.8 ± 2.82	Weak	MTT	[46]
Isocoronarin D **(17)** 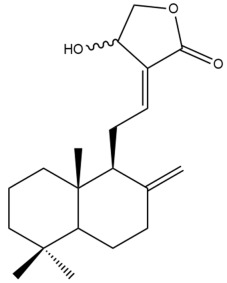	IC_50_: 17.3	Moderate	MTT	[37]
6-oxo-7,11,13-labdatrien-17-al-16,15-olide **(18)** 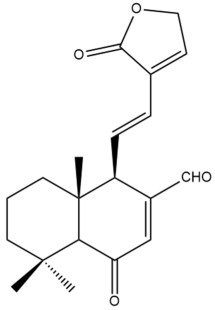	GI_50_: 18.5 ± 0.95	Moderate	SRB	[36]
7,17-dihydroxy-6-oxo-7,11,13-labdatrien-16,15-olide **(19)** 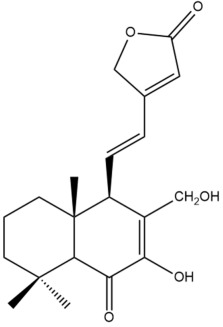	GI_50_: 26 ± 1.7	Moderate	SRB	[36]
Coronarin D **(20)** 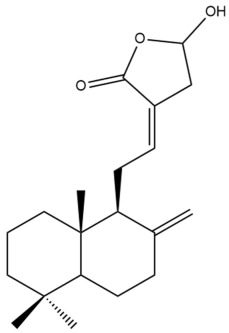	GI_50_: 8 ± 0.95	**Strong**	SRB	[36]
	IC_50_: 86.42	Weak	MTT	[37]
Coronarin C **(21)** 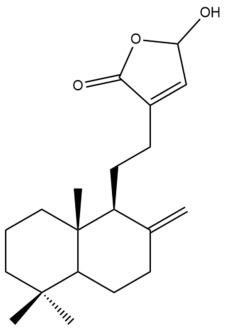	GI_50_: 4.8 ± 0.19	**Strong**	SRB	[36]
Hedychenone **(22)** 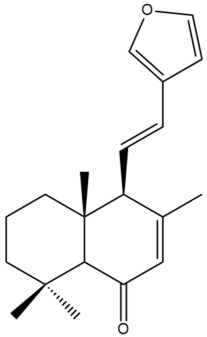	GI_50_: 7.4 ± 0.65	**Strong**	SRB	[36]
6-oxo-7,11,13-labdatriene-16,15-olide **(23)** 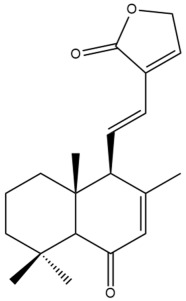	GI_50_: 21.7 ± 0.44	Moderate	SRB	[36]
Pacovatinin A **(24)** 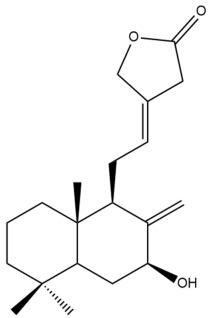	GI_50_: 15.1 ± 0.5	Moderate	SRB	[36]
Coronarin B **(25)** 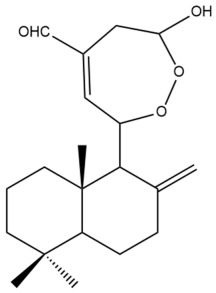	IC_50_: 85.39	Weak	MTT	[37]
Coronarin E **(27)** 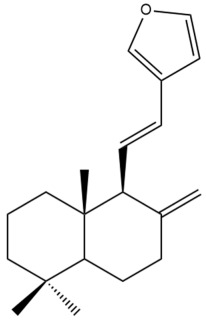	IC_50_: 53.26 ± 1.09	Weak	MTT	[43]
Yunnancoronarin A **(32)** 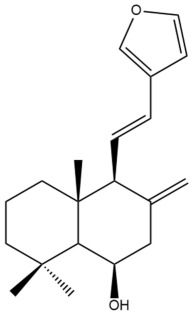	IC_50_: 11.08	Moderate	MTT	[40]
Yunnancoronarin B **(33)** 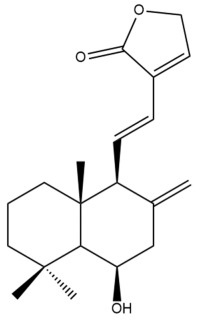	IC_50_: 0.92	**Potent**	MTT	[40]
7-hydroxy,15,16-epoxy-17-al-7,11,13(16),14-labda-tetraene-6-one (7-hydroxy hedichinal) **(37)** 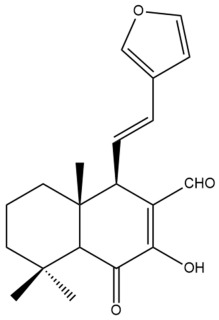	IC_50_: 37.85 ± 1.01	Weak	MTT	[43]
14,15,16-trinor-7,11-labdadien-13-oicacid (spicatanoic acid) **(38)** 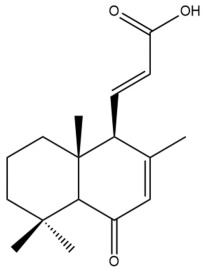	IC_50_: 51.97 ± 1.09	Weak	MTT	[43]
Yunnancoronarin D **(39)** 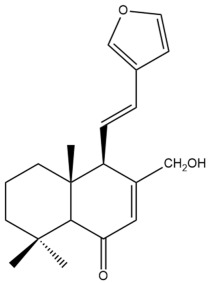	IC_50_: 51.24 ± 1.21	Weak	MTT	[43]
Coronarin K **(43)** 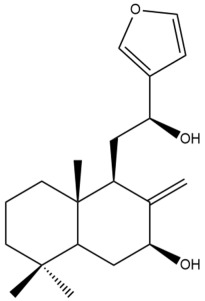	IC_50_: 13.49 ± 0.62	Moderate	MTT	[46]
Coronarin L **(44)** 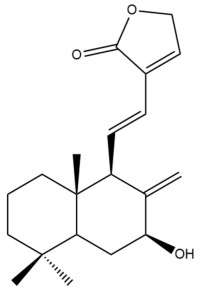	IC_50_: 33.78 ± 1.37	Weak	MTT	[46]

**Table 19:** Cytotoxic activities of labdane-type diterpenes from the Zingiberaceae against NCI-H187 lung cancer cell line.

Compound	IC_50_/GI_50_ (µM)		Assay	Reference
	NCI-H187	Potency		
Zerumin B **(6)** 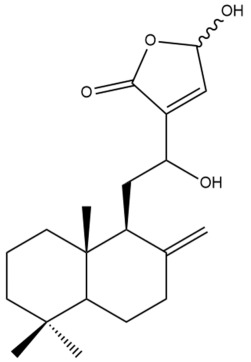	IC_50_: 18.36	Moderate	REMA	[39]
Coronarin A **(14)** 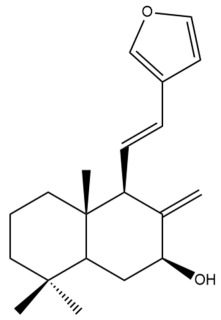	IC_50_: 40.77	Weak	REMA	[41]
16-Hydroxylabda-8(17),11,13-trien-15,16-olide **(16)** 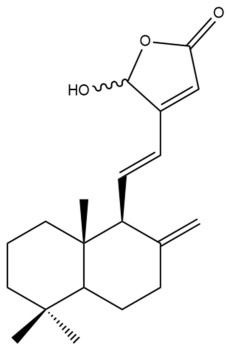	IC_50_: 2.18	**Strong**	REMA	[39]
Coronarin D **(20)** 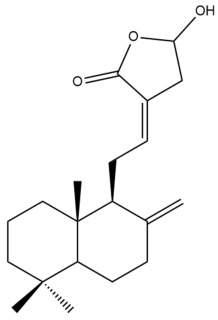	IC_50_: 80.77	Weak	REMA	[39]
Coronarin E **(27)** 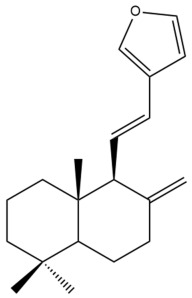	IC_50_: 63.50	Weak	REMA	[39]
	IC_50_: 49.73	Weak	REMA	[41]
(*E*)-15,16-Bisnorlabda-8(17),11-dien-13-one **(28)** 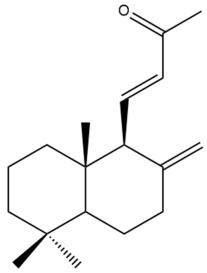	IC_50_: 83.22	Weak	REMA	[39]
Villosin **(29)** 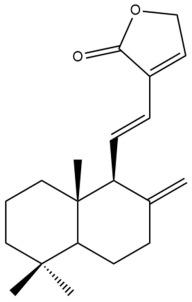	IC_50_: 0.40	**Potent**	REMA	[39]
	IC_50_: 0.40	**Potent**	REMA	[41]
15-Methoxylabda-8(17),11,13-trien-15,16-olide **(30)** 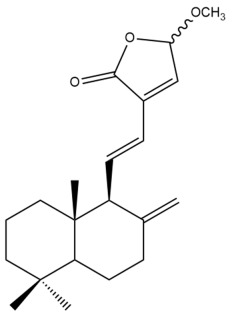	IC_50_: 2.72	**Strong**	REMA	[39]
(*E*)-labda-8(17),12-dien-15,16-dial **(31)** 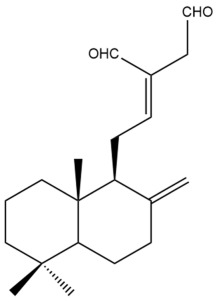	IC_50_: 36.93	Weak	REMA	[39]
Yunnancoronarin A **(32)** 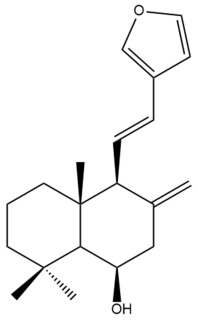	IC_50_: 36.78	Weak	REMA	[41]
Yunnancoronarin B **(33)** 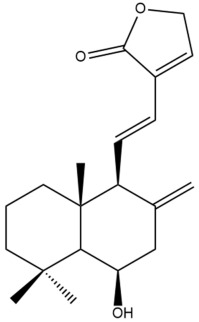	IC_50_: 44.57	Weak	REMA	[41]
Hedyforrestin B **(34)** 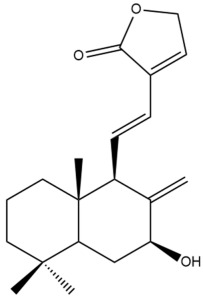	IC_50_: 3.10	**Strong**	REMA	[41]
Hedyforrestin C **(35)** 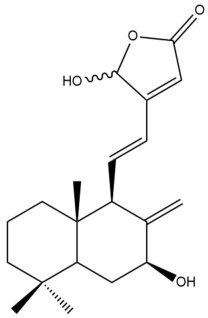	IC_50_: 2.46	**Strong**	REMA	[41]

**Table 20:** Cytotoxic activities of labdane-type diterpenes from the Zingiberaceae against selected prostate cancer cell lines.

Compound	IC_50_ (µM)		Assay	Reference
	DU145	Potency		
Zerumin B **(6)** 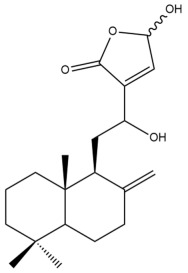	IC_50_: 11.21 ± 3.1 µM	Moderate	MTT	[33]

## Data Availability

Not applicable.

## Supplementary Materials

The following supporting information can be downloaded at: https://www.mdpi.com/article/10.3390/ph15121517/s1, Figure S1: Cytotoxic labdane type diterpenes from the Zingiberaceae; Figure S2. Labdane framework; Table S1: Extraction, isolation, and characterisation of cytotoxic labdane type diterpenes from the Zingiberaceae.

## Abbreviations and Acronyms

A-549	Lung adenocarcinoma
BxPC-3	Pancreatic ductal adenocarcinoma
COLO 205	Colon adenocarcinoma
DLD-1	Colon adenocarcinoma
DU145	Prostate carcinoma
GI_50_	Half-maximal inhibition of cell proliferation
GLOBOCAN	Global Cancer, Incidence, Mortality and Prevalence
HCC-S102	Hepatocellular carcinoma
HCT-116	Colon carcinoma
HeLa	Human papillomavirus-related endocervical adenocarcinoma
HepG2	Hepatoblastoma
HPLC	High performance liquid chromatography
HT-29	Colon adenocarcinoma
IC_50_	Half-maximal inhibitory concentration
IR	Infrared spectroscopy
KB	Human papillomavirus-related endocervical adenocarcinoma
MCF-7	Michigan Cancer Foundation-7 (breast cancer)
MDA-MB-231	MD Anderson-Metastatic Breast-231 (triple-negative breast cancer)
MS	Mass spectrometry
MTT	3-[4,5-dimethylthiazol-2-yl]-2,5 diphenyl tetrazolium bromide
NCI-H187	Lung small-cell carcinoma
NMR	Nuclear magnetic resonance spectroscopy
PICO	Population, Intervention, Comparison and Outcome
PRISMA	Preferred Reporting Items for Systematic Reviews and Meta-Analyses
REMA	Resazurin microtiter assay
SAR	Structure–activity relationship
SGC-7901	Human papillomavirus-related endocervical adenocarcinoma
SMMC-7721	Human papillomavirus-related endocervical adenocarcinoma
SRB	Sulforhodamine B
T-47D	Breast cancer
TLC	Thin-layer chromatography
UV-Vis	Ultraviolet–visible spectroscopy
WHO	World Health Organization
