# Supplementary material for: Cytotoxic Labdane Diterpenes, Norlabdane Diterpenes and Bis-Labdanic Diterpenes from the Zingiberaceae: A Systematic Review
Source: Pharmaceuticals (Basel). 2022 Dec 5;15(12):1517. doi: 10.3390/ph15121517 (PMC9783331; doi:10.3390/ph15121517)
Supplement: Supplementary file 1 [file pharmaceuticals-15-01517-s001.zip › pharmaceuticals-2001606-supplementary.pdf]

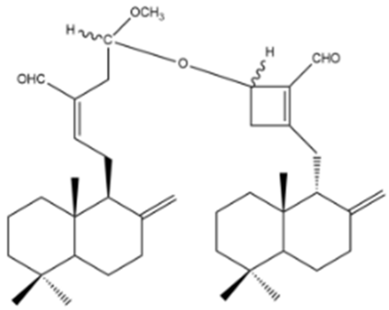

1

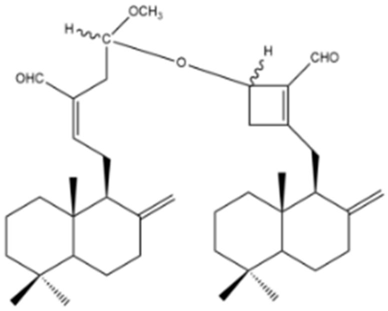

2

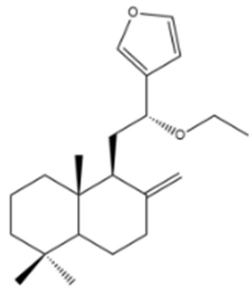

3

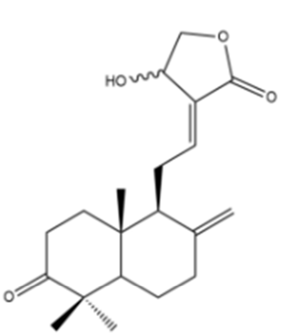

4

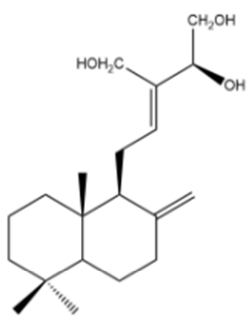

5

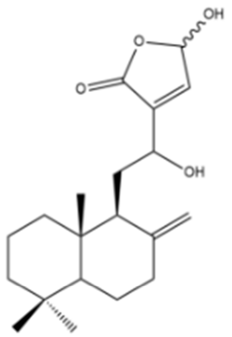

6

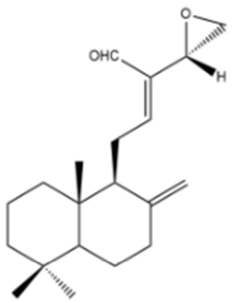

7

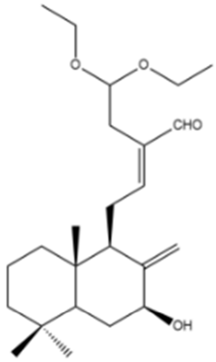

8

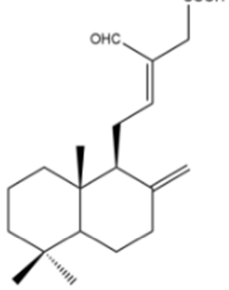

9

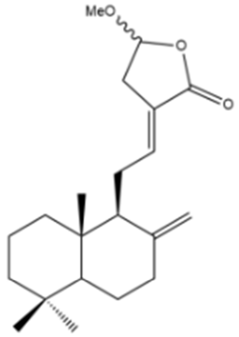

10

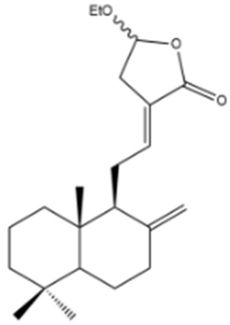

11

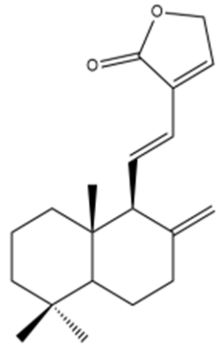

12

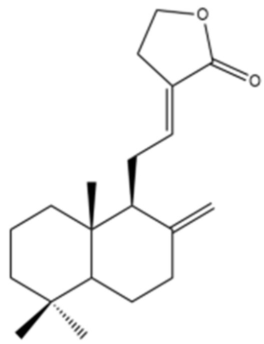

13

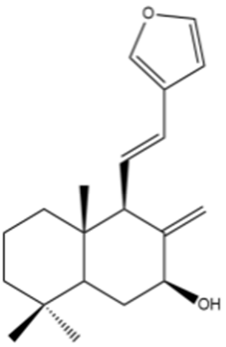

14

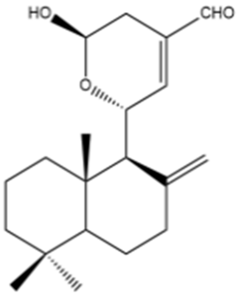

15

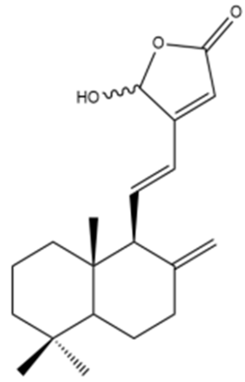

16

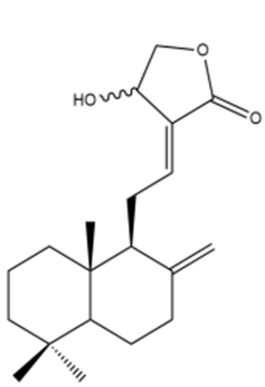

17

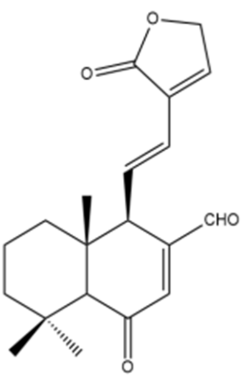

18

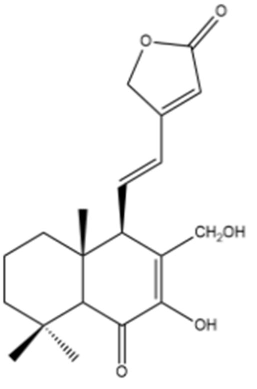

19

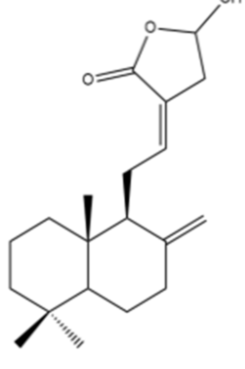

20

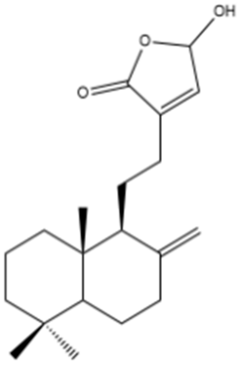

21

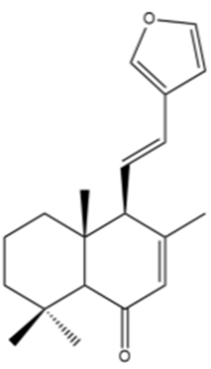

22

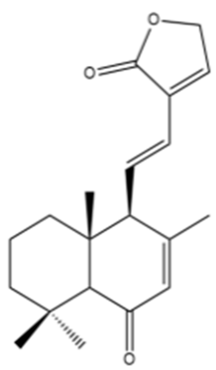

23

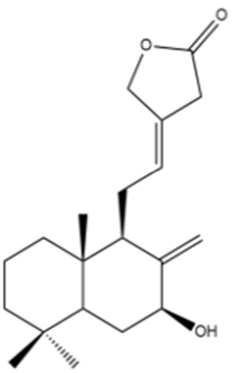

24

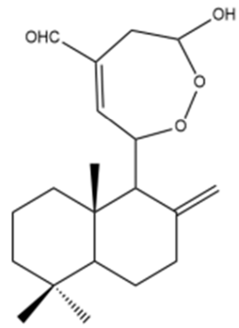

25

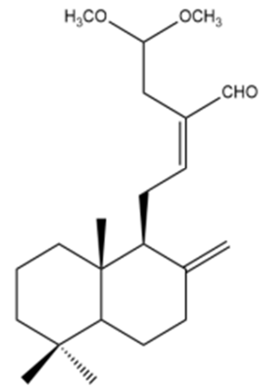

26

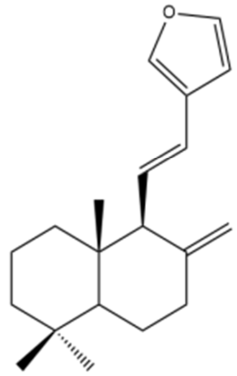

27

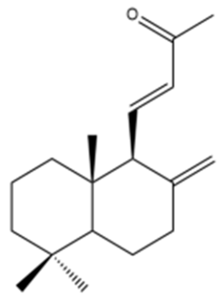

28

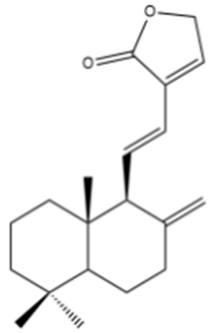

29

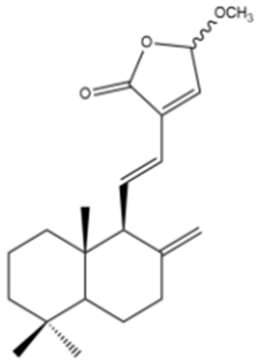

30

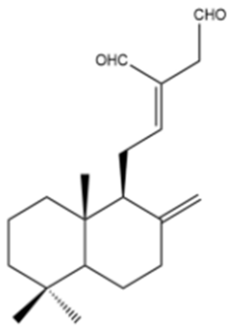

31

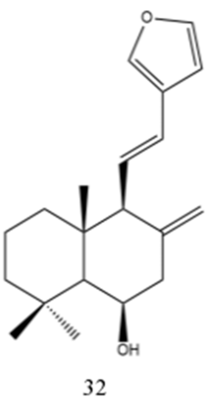

32

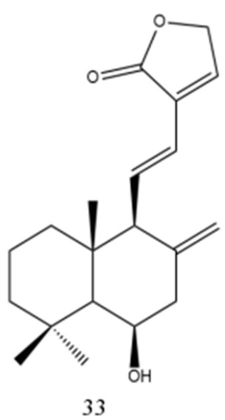

33

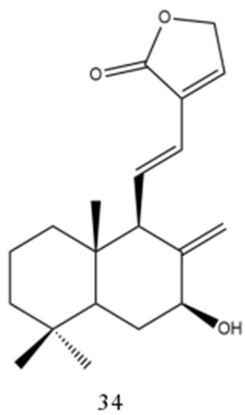

34

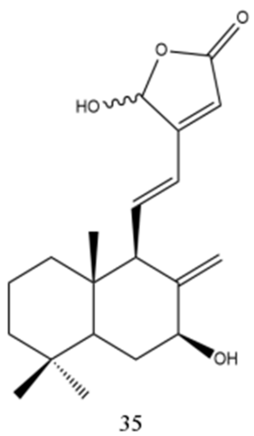

35

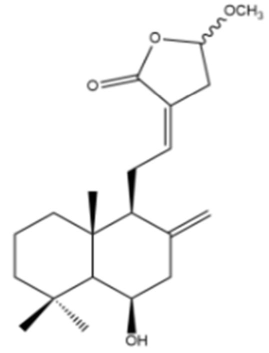

36

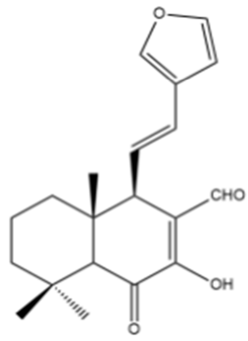

37

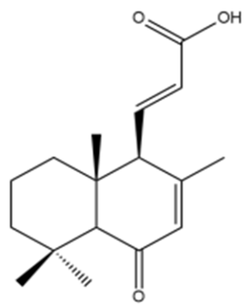

38

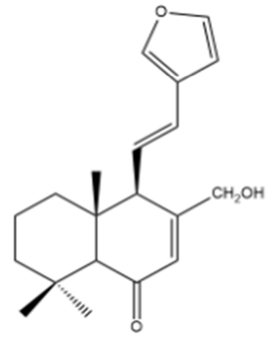

39

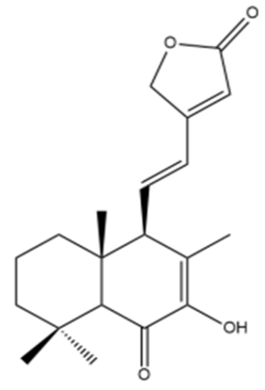

40

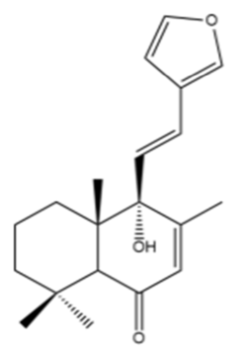

41

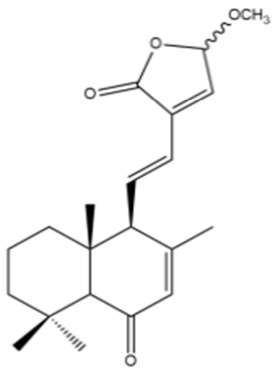

42

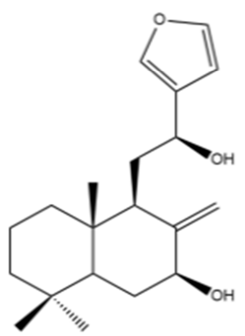

43

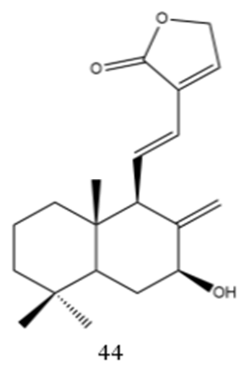

44

Figure S1. Cytotoxic labdane type diterpenes from the Zingiberaceae

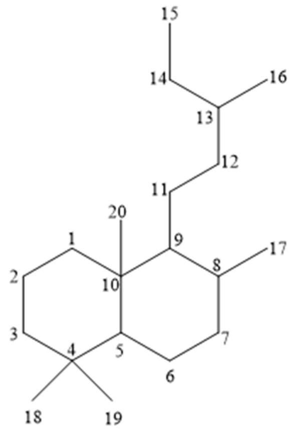

Figure S2. Labdane framework

Table S1. Extraction, isolation and characterisation of cytotoxic labdane type diterpenes from the Zingiberaceae

Species	Part of the species investigated	Method of extraction	Method of isolation	Method of characterisation	Name of labdane type diterpenes	Reference
Alpinia calcarata Rosc.	Rhizomes	Extracted with methanol<br><br>Methanolic extract was partitioned with ethyl acetate	Column chromatography (silica gel)<br><br>Preparative TLC<br><br>Preparative HPLC	IR, UV, HRFABMS, 1D and 2D NMR	Calcaratarin D (**1**)<br><br>Calcaratarin E (**2**)	[30]
Alpinia intermedia Gagnep	Leaves	Refluxed with 95% ethanol<br><br>Aqueous ethanolic extract was partitioned with hexane	Column chromatography (silica gel or flash silica gel)	GC-MS, 1D and 2D NMR NMR, Single Crystal X-Ray	Intermedin A (**3**)	[31]
Amomum maximum Roxb	Roots	Extracted with 95% ethanol<br><br>Aqueous ethanolic extract was partitioned with	Column chromatography (silica gel/Sephadex LH-20/ODS)	IR, UV, HR-ESI-MS, [1]1D and 2D NMR	Amomax C (**4**)<br><br>(12*Z*,14*R*)-labda-8(17),12- diene-14,15,16-triol (**5**)	[32]

		dichloromethane	Preparative HPLC (RP-C18 column)			
Curcuma mangga Val.van Zip	Rhizomes	Extracted with acetone<br><br>Acetone extract was partitioned with hexane followed by ethyl acetate	Column chromatography (silica gel)	IR, UV, HREIMS, ^{1}1D and 2D NMR	Zerumin B (**6**)	[33]
Curcuma mutabilis Škorničk., M. Sabu & Prasanthk.	Rhizomes	Extracted with petroleum ether	Column chromatography (silica gel)	IR, HR-ESI-MS, ^{1}1D and 2D NMR	(*E*)-14,15-epoxylabda-8(17),12-dien-16-al (Cm epoxide) (**7**)	[34]
Hedychium coronarium J. Koenig	Rhizomes	Extracted with 95% EtOH<br><br>Aqueous ethanolic extract was partitioned with ethyl acetate	Column chromatography (silica gel)	IR, UV, HR-ESI-MS, ^{1}1D and 2D NMR	Hedycoronal A (**8**)<br><br>Hedycoronal B (**9**)<br><br>Coronarin D methyl ether (**10**)<br><br>Coronarin D ethyl ether (**11**)<br><br>Labda-8(17),11,13-trien-15(16)-olide (**12**)<br><br>(12*E*)-Labda-8(17),12-dien-15(16)-olide (**13**)<br><br>Coronarin A (**14**)<br><br>15-Hydroxy-11,15-epoxylabda-8(17),12-dien-16-al (**15**)	[35]

					16-Hydroxylabda-8(17),11,13-trien-15,16-olide (**16**)<br><br>Isocoronarin D (**17**)	
Hedychium coronarium J. Koenig	Rhizomes	Soxhlet extraction with hexane	Column chromatography (silica gel)<br><br>Preparative TLC	IR, HR-ESI-MS, 11D and 2D NMR	Coronarin D methyl ether (**10**)<br><br>6-oxo-7,11,13-labdatrien-17-al-16,15-olide (**18**)<br><br>7,17-dihydroxy-6-oxo-7,11,13-labdatrien-16,15-olide (**19**)<br><br>Coronarin D (**20**)<br><br>Coronarin C (**21**)<br><br>Hedychenone (**22**)<br><br>6-oxo-7,11,13-labdatriene-16,15-olide (**23**)<br><br>Pacovatinin A (**24**)	[36]
Hedychium coronarium J. Koenig	Rhizomes	Macerated with dichloromethane	Vacuum liquid chromatography (silica gel)<br><br>Column chromatography (silica gel)	IR, UV, HRMS (ESI-TOF), 1D and 2D NMR	Isocoronarin D (**17**)<br><br>Coronarin D (**20**)<br><br>Coronarin B (**25**)	[37]
Hedychium coronarium J. Koenig	Aerial parts	Extracted with MeOH<br><br>Methanolic extract was partitioned with hexane	Column chromatography (silica gel)<br><br>Preparative TLC	IR, UV, HR-ESI-MS, 1D and 2D NMR	Coronarin A (**14**)<br><br>Calcaratarin A (**26**)	[38]

Hedychium ellipticum Buch-Ham. ex Sm.	Rhizomes	Extracted with Hexane followed by dichloromethane	Column chromatography (silica gel/Sephadex LH-20)	IR, HREIMS (Q-TOF), 1D and 2D NMR	Zerumin B (**6**)<br>16-Hydroxylabda-8(17),11,13-trien-15,16-olide (**16**)<br>Coronarin D (**20**)<br>Coronarin E (**27**)<br>(*E*)-15,16-Bisnorlabda-8(17),11-dien-13-one (**28**)<br>Villosin (**29**)<br>15-Methoxylabda-8(17),11,13-trien-15,16-olide (**30**)<br>(*E*)-labda-8(17),12-dien-15,16-dial (**31**)	[39]
Hedychium forrestii Tong.	Rhizomes	Extracted with hot 95% ethanol<br>Ethanolic extract was partitioned with ethyl acetate	Column chromatography (silica gel)	IR, HR-EI-MS, 1D and 2D NMR	Yunnancoronarin A (**32**)<br>Yunnancoronarin B (**33**)	[40]
Hedychium gardnerianum Sheppard ex Ker Gawl	Rhizomes	Successively extracted with hexane and dichloromethane	Column chromatography (silica gel)	IR, EIMS, 1D and 2D NMR	Coronarin A (**14**)<br>Coronarin E (**27**)<br>Villosin (**29**)<br>Yunnancoronarin A (**32**)<br>Yunnancoronarin B (**33**)<br>Hedyforrestin B (**34**)	[41]

					Hedyforrestin C (**35**)	
Hedychium longipetalum X. Hu & N. Liu	Rhizomes	N/A	N/A	IR, UV, HR-ESI-MS, 1D and 2D NMR	Yunnancoronarin A (**32**)<br><br>Hedyforrestin B (**34**)<br><br>Hedyforrestin C (**35**)<br><br>Hedylongnoid C (**36**)	[42]
Hedychium spicatum Buch.-Ham. ex Sm.	Rhizomes	Extracted with dichloromethane/methanol (1:1)	Column chromatography (silica gel)<br><br>Preparative HPLC (C18 column)	IR, HR-ESI-MS, 1D and 2D NMR	Coronarin E (**27**)<br><br>7-hydroxy,15,16-epoxy-17-al-7,11,13(16),14-labda-tetraene-6-one (7-hydroxy hedichinal) (**37**)<br><br>14,15,16-trinor-7,11-labdadien-13-oicacid (spicatanoic acid) (**38**)<br><br>Yunnancoronarin D (**39**)	[43]
Hedychium spicatum Buch.-Ham. ex Sm.	Rhizomes	Soxhlet extraction with chloroform	Column chromatography (silica gel)	IR, HR-ESI-MS, 1D and 2D NMR	Yunnancoronarin A (**32**)<br><br>7-hydroxy,6-oxo-7,11,13-labdatrien-16,15-olide (hedychilactone D) (**40**)<br><br>9-hydroxy,15,16-epoxy-7,11,13(16)14-labdatetraen-6-one (9-hydroxy hedychenone) (**41**)	[44]
Hedychium yunnanense Gagnep.	Rhizomes	Macerated with ethanol under reflux<br><br>Ethanolic extract was partitioned	Column chromatography (Silica gel/MCI gel)<br><br>Semipreparative HPLC (RP-C18)	IR, UV, HR-ESI-MS, 1D and 2D NMR	Hedychenone (**22**)<br><br>Villosin (**29**)<br><br>Hedychenoid B (**42**)	[45]

		with ethyl acetate				
Roscoea purpurea Sm.	Rhizomes	Extracted with methanol<br><br>Methanolic extract was partitioned with petroleum ether followed by chloroform	Column chromatography (Silica gel)	IR, UV, HR-ESI-MS, 1D and 2D NMR	Coronarin A (**14**)<br><br>Coronarin K (**43**)<br><br>Coronarin L (**44**)	[46]
